# Understanding the constitutive presentation of MHC class I immunopeptidomes in primary tissues

**DOI:** 10.1016/j.isci.2022.103768

**Published:** 2022-01-18

**Authors:** Peter Kubiniok, Ana Marcu, Leon Bichmann, Leon Kuchenbecker, Heiko Schuster, David J. Hamelin, Jérôme D. Duquette, Kevin A. Kovalchik, Laura Wessling, Oliver Kohlbacher, Hans-Georg Rammensee, Marian C. Neidert, Isabelle Sirois, Etienne Caron

**Affiliations:** 1 CHU Sainte-Justine Research Center, Montreal, QC H3T 1C5, Canada; 2 Department of Immunology, Interfaculty Institute for Cell Biology, University of Tübingen, 72076 Tübingen, Baden-Württemberg, Germany; 3 Cluster of Excellence iFIT (EXC 2180), “Image-Guided and Functionally Instructed Tumor Therapies”, University of Tübingen, 72076 Tübingen, Baden-Württemberg, Germany; 4 Applied Bioinformatics, Department of Computer Science, University of Tübingen, 72074 Tübingen, Baden-Württemberg, Germany; 5 Immatics Biotechnologies GmbH, 72076 Tübingen, Baden-Württemberg, Germany; 6 DKFZ Partner Site Tübingen, German Cancer Consortium (DKTK), 72076 Tübingen, Baden-Württemberg, Germany; 7 Institute for Bioinformatics and Medical Informatics, University of Tübingen, 72076 Tübingen, Baden-Württemberg, Germany; 8 Biomolecular Interactions, Max Planck Institute for Developmental Biology, 72076 Tübingen, Baden-Württemberg, Germany; 9 Cluster of Excellence Machine Learning in the Sciences (EXC 2064), University of Tübingen, 72074 Tübingen, Baden-Württemberg, Germany; 10 Translational Bioinformatics, University Hospital Tübingen, 72076 Tübingen, Baden-Württemberg, Germany; 11 Clinical Neuroscience Center and Department of Neurosurgery, University Hospital and University of Zürich, 8057&8091 Zürich, Switzerland; 12 Department of Pathology and Cellular Biology, Faculty of Medicine, Université de Montréal, QC H3T 1J4, Canada

**Keywords:** Biological sciences, Biomolecules, Immunology, Peptides

## Abstract

Understanding the molecular principles that govern the composition of the MHC-I immunopeptidome across different primary tissues is fundamentally important to predict how T cells respond in different contexts *in vivo*. Here, we performed a global analysis of the MHC-I immunopeptidome from 29 to 19 primary human and mouse tissues, respectively. First, we observed that different HLA-A, HLA-B, and HLA-C allotypes do not contribute evenly to the global composition of the MHC-I immunopeptidome across multiple human tissues. Second, we found that tissue-specific and housekeeping MHC-I peptides share very distinct properties. Third, we discovered that proteins that are evolutionarily hyperconserved represent the primary source of the MHC-I immunopeptidome at the organism-wide scale. Fourth, we uncovered new components of the antigen processing and presentation network, including the carboxypeptidases CPE, CNDP1/2, and CPVL. Together, this study opens up new avenues toward a system-wide understanding of antigen presentation *in vivo* across mammalian species.

## Highlights

• Tissue-specific and housekeeping MHC class I peptides share distinct properties
• HLA-A, HLA-B, and HLA-C allotypes contribute very unevenly to the pool of class I peptides
• MHC-I immunopeptidomes are represented by evolutionarily conserved proteins
• An extended antigen processing and presentation pathway is uncovered

## Graphical abstract

**Figure:**
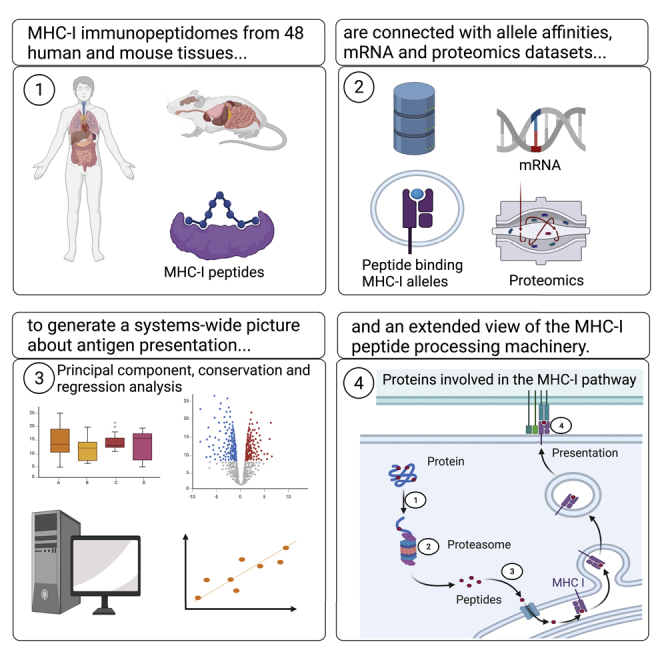

## Introduction

In adaptive immunity, CD8^+^ T cells have the ability to eradicate abnormal cells through recognition of small peptide fragments presented by MHC (human leukocyte antigen (HLA) in humans) class I molecules. In this context, jawed vertebrates evolved an important antigen processing and presentation (APP) system capable of presenting thousands of different MHC class I peptides on the surface of virtually any nucleated cells (Neefjes et al., 2011), and transmissible tumors could be a selective factor of APP evolution (Dujon et al., 2020; Gastaldello et al., 2021). In mammals, around 200 different cell types are decorated by large repertoires of self-MHC-I-associated peptides, collectively referred to as the mammalian MHC-I immunopeptidome (MHC-I immunopeptidome) (Caron et al., 2017; Vizcaíno et al., 2020).

The interindividual and intraindividual complexity of the MHC-I immunopeptidome accounts for its overall heterogeneity (Gfeller and Bassani-Sternberg, 2018; Maccari et al., 2017; Vizcaíno et al., 2020). In fact, each MHC-I allotype generally presents a distinct subset of peptide antigens, which are characterized by the presence of specific anchor residues that are necessary to bind MHC-I (Falk et al., 1991). In human, up to six different HLA-I allotypes are expressed at the individual level, and thousands, if not millions of different HLA-I allotypes are expressed across human populations, hence increasing enormously the inter-individual heterogeneity of the MHC-I immunopeptidome (Robinson et al., 2017). In contrast, the MHC-I immunopeptidome of the C57BL/6 mouse strain is relatively simpler because peptide antigens are presented by only two classical MHC-I molecules (H2D^b^ and H2K^b^). In addition to its allotype-dependent composition, the mammalian MHC-I immunopeptidome is also complicated by its tissue-dependency. In fact, two pioneering mapping studies recently pointed toward large variations in the repertoire of MHC-I-associated peptides across different tissues (Marcu et al., 2021; Schuster et al., 2018). However, very little is known about the molecular principles that shape the tissue-dependent processing and presentation of peptide antigens at the organism level.

Classical biochemistry approaches have established the blueprint of antigen processing and presentation (Neefjes et al., 2011; Yewdell et al., 2003). In a nutshell, the biogenesis of peptides presented by MHC-I molecules is initiated with the transcription and translation of the source genes, and the resulting proteins are typically degraded by the proteasome and/or immunoproteasome in the nucleus and cytosol (Kincaid et al., 2011). Cytosolic peptides are rapidly targeted by cytosolic aminopeptidases, such as thimet oligopeptidase (TOP) (York et al., 2003), leucine aminopeptidase (LAP) (Towne et al., 2005), and tripeptidyl peptidase II (TPPII) (Reits et al., 2004), which trim and destroy most peptides. A fraction of peptides escapes destruction by translocation into the ER lumen via transporter associated with antigen presentation (TAP) (Reits et al., 2000; Yewdell et al., 2003). In the ER, peptides may be further trimmed by ER aminopeptidase associated with antigen processing (ERAAP) and then bind MHC-I molecules for stabilization by the peptide loading complex (Blees et al., 2017; Serwold et al., 2002). Once stable, MHC-I-peptide complexes are released from the ER and are transported to the cell surface for peptide presentation to CD8^+^ T cells.

Modern immunopeptidomics is driven by high-resolution mass spectrometry (MS) and investigates the composition and dynamics of the MHC-I immunopeptidome (Caron et al., 2015a; 2015b). Complementing classical biochemistry techniques, immunopeptidomic technology platforms have yielded important systematic insights into the biogenesis of the MHC-I immunopeptidome (Granados et al., 2015). For instance, they have refined binding motifs for a wide range of MHC-I alleles in human (Abelin et al., 2017; Gfeller and Bassani-Sternberg, 2018), they have indicated that large numbers of MHC-I peptides derive from genomic ‘hotspots’ (Müller et al., 2017; Pearson et al., 2016) as well as noncoding genomic regions (Laumont et al., 2018), and they have demonstrated that abundant transcripts and proteins contribute preferentially to the composition of the MHC-I immunopeptidome (Abelin et al., 2017; Bassani-Sternberg et al., 2015; Fortier et al., 2008; Granados et al., 2012; Pearson et al., 2016). Furthermore, immunopeptidomic approaches have validated that defective ribosomal products (DRiPs), immunoproteasome subunits as wells as other key players involved in the processing of peptide antigens (e.g., proteasome, ERAAP) markedly influence the repertoire of peptides presented by MHC-I molecules (Bourdetsky et al., 2014; Milner et al., 2013; Nagarajan et al., 2016; Trentini et al., 2020; Verteuil et al., 2010).

The understanding of how the MHC-I immunopeptidome is generated in different primary tissues *in vivo*, in human as well as in animal models, is fundamentally important to rationalize and predict how T cells respond in various contexts (Tscharke et al., 2015). However, immunopeptidomics studies that focused on the systematic deciphering of the MHC-I immunopeptidome biogenesis have been almost exclusively conducted in transformed cells. Therefore, the rules that govern the composition and tissue-dependency of the mammalian MHC-I immunopeptidome remain poorly understood and many fundamental questions remain unanswered to date. For instance, what is the relative contribution of individual HLA-I allotypes to the composition of the MHC-I immunopeptidome within and across tissues? To what extent does the MHC-I immunopeptidome conceal tissue-specific patterns/signatures that are conserved across species? What are the many transcription factors, proteases, aminopeptidases, and carboxypeptidases involved in the generation and processing of MHC-I peptides in different tissues, and how does the expression and activity of those proteins influence the tissue-dependency and overall heterogeneity of the MHC-I immunopeptidome at the organism-wide scale? In this study, we applied a systems-level, cross-species approach to tackle these fundamental questions.

## Results

**Figure 1:**
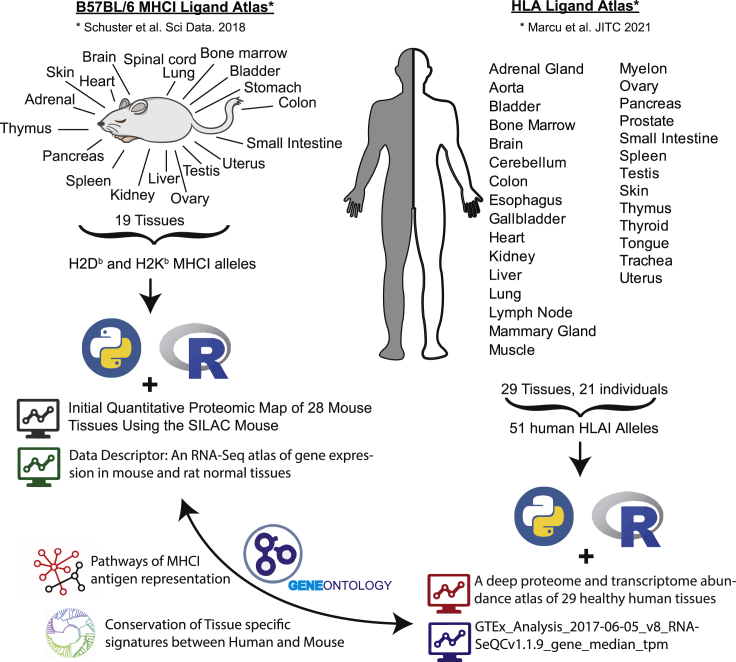
Overview of immunopeptidomics, proteomics, and transcriptomics datasets analyzed (**Left hand side**) Graphic description of the Mouse B57BL/6 MHCI Ligand Atlas, which was connected with two published proteomics and mRNA expression atlases of mouse tissues. (**Right hand side**) Graphic description of the Human HLAI Ligand Atlas, which was connected with two published proteomics and mRNA expression atlases of human tissues

Two immunopeptidomic mapping studies have very recently drafted the first tissue-based atlases of the mouse and human MHC-I immunopeptidome (Marcu et al., 2021; Schuster et al., 2018). These pioneering mapping efforts provide qualitative and semiquantitative information about the currently detectable repertoire of MHC-I peptides in most organs, both in mouse and human. Specifically, the mouse atlas was generated from 19 normal primary tissues extracted from C57BL/6 mice expressing H2K^b^ and H2D^b^ (Schuster et al., 2018). The human atlas was generated from 29 human benign tissues extracted from 21 different subjects expressing a total of 51 different HLA-I allotypes (Marcu et al., 2021) (Figure 1). Those HLA-I allotypes cover the most frequent HLA-A, HLA-B, and HLA-C alleles in the world. Below, we first focused on the analysis of the MHC-I immunopeptidome in different mouse and human tissues to provide a general understanding of the heterogeneity, tissue-dependency, and conservation patterns of the MHC-I immunopeptidome. Next, we connected tissue immunopeptidomes to RNA-seq and protein expression data found in various tissue-based atlases (Geiger et al., 2013; Söllner et al., 2017; Wang et al., 2019) to dissect how the mammalian MHC-I immunopeptidome is being shaped in different tissues (Figure 1).

### HLA-I allotypes are unevenly represented across tissue immunopeptidomes

**Figure 2:**
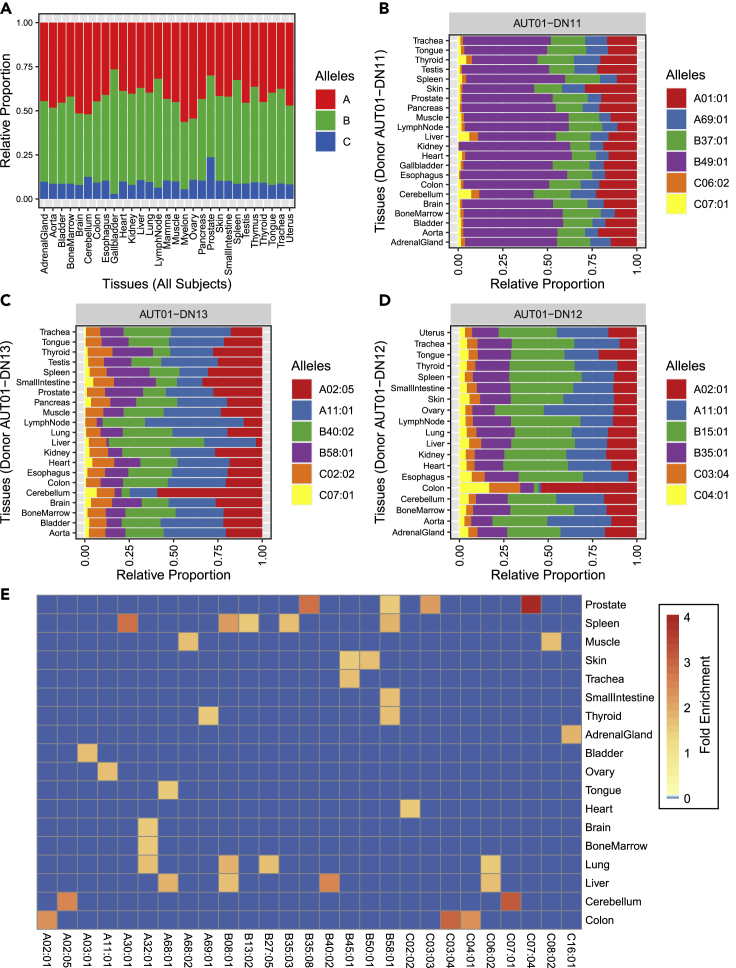
Distribution of HLA-A-specific, B-specific, and C-specific immunopeptidomes across human tissues (A) Relative proportion of individual HLA-A-specific, B-specific, and C-specific immunopeptidomes per tissue among all subjects. (B–D) Relative proportion of individual HLA allele-specific immunopeptidomes per tissue for AUT-DN11 (B), AUT-DN13 (C), and AUT-DN12 (D). (E) Enrichment of HLAI allotypes across all tissues sampled. Average enrichment values are depicted where allotypes were sampled across several subjects

A key open question regarding the heterogeneity of the human MHC-I immunopeptidome is whether individual HLA-I allotypes contribute evenly or unevenly to the composition of the MHC-I immunopeptidome across different tissues. In fact, every subject presents up to two HLA-A, two HLA-B, and two HLA-C allotypes. If all allotypes were evenly represented at the cell surface across tissues, one would expect similar proportions of peptides assigned to each allotype in all tissues. To address this question, we first assessed the global tissue distribution of all detectable peptides that were assigned to HLA-A, HLA-B, and HLA-C. Among 29 sampled benign tissues extracted from a total of 21 different subjects, we found HLA-A, HLA-B, and HLA-C immunopeptidomes to be unevenly represented across tissues (Figures 2A and S1A). To increase the resolution of this analysis, we investigated the contribution of each HLA-A, HLA-B, and HLA-C allotypes expressed in the three subjects for which the most tissues had been sampled (i.e., AUT-DN11, AUT-DN13, and AUT-DN12) (Figures 2B–2D). Consistently, we found differential peptide distributions across tissues for many HLA-I allotypes. For instance, ∼55% of peptides in the Colon of subject AUT-DN12 were assigned to A∗02:01 compared to ∼22% on average in all other tissues, resulting in an enrichment of about 2.5-fold for A∗02:01 (Figure 2D). The enrichment of A∗02:01 peptides in the Colon of subject AUT-DN12 was also further accompanied by an underrepresentation of A∗11:01, B∗15:01, and B∗35:01 in the Colon, and an enrichment of C∗03:04 and C∗04:01 alleles (Figure 2D). Similarly, we also noted that ∼50% of peptides in the liver of subject AUT-DN13 were assigned to HLA-B40:02 compared to ∼20% on average in all other tissues, resulting in an enrichment of about 2.5-fold for this specific HLA-B allotype in this particular subject (Figure 2C). To provide a global picture about enrichment values that are associated with individual HLA-I allotypes, we calculated the average enrichment of all HLA-I allotypes across the investigated subjects and highlighted alleles that were enriched by more than 1.5-fold in at least one tissue (Figure 2E). This analysis highlighted 37 enrichment values distributed across 27 specific HLA-I allotypes and 18 different tissues (Figure 2E). Overall, those enrichment values ranged from 1.5 to 8.1-fold, and nine (out of 16) HLA-A, 9 (out of 21) HLA-B, and 9 (out of 14) HLA-C allotypes were assigned in at least one tissue with an enrichment value above 1.5-fold. Thus, our results show that HLA-I allotypes do not contribute evenly to the composition of the MHC-I immunopeptidome across different tissues and subjects, and therefore, considerably contribute to the overall heterogeneity of the human MHC-I immunopeptidome.

### The high level of heterogeneity among immunopeptidomes of different tissues shows pronounced similarities between mouse and human

**Figure 3:**
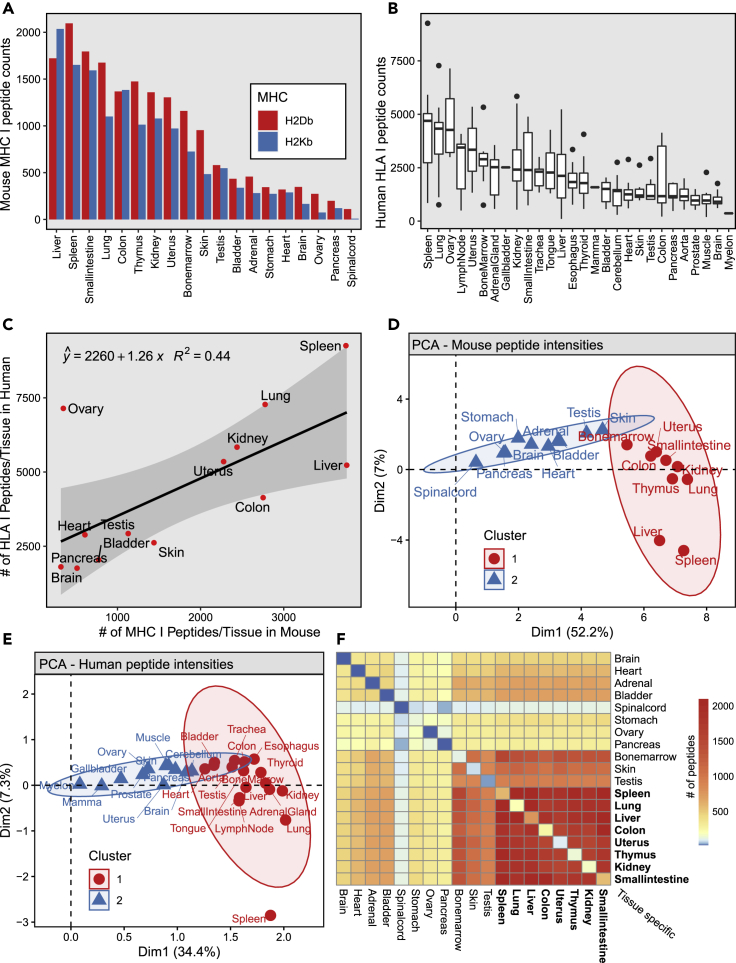
Comparison of tissue dependent MHCI-associated (Mouse) and HLAI (Human) -associated peptides (A) MHCI peptide counts for each sampled mouse tissue, colors depict the MHCI alleles (H2D^b^ and H2K^b^). (B) HLAI peptide counts for all sampled human tissues. Boxplots are represented as several tissues were sampled across different individuals. (C) Comparison of MHCI peptide counts/tissue (Mouse) and HLAI peptide counts/tissue (Human). (D) Principal component analysis of the measured intensities (log10) of MHCI peptides (Mouse). (E) Principal component analysis of the measured intensities (log10) of HLAI peptides (Human). (F) Tissue connectivity map of the ‘B57BL/6 MHCI Ligand atlas. Heatmap depicts the number of shared MHCI peptides across tissues (Mouse). Note: The number of uniquely observed/tissue-specific peptides can be found along the diagonal. Spinal cord (mouse) and Myelon (human) are equivalent terms

Antigen processing and presentation is a conserved and ubiquitous biological process in mammals. Here, we hypothesized that the MHC-I immunopeptidome of different tissues might conceal tissue-dependent immunopeptidomic patterns/signatures that are conserved between mouse and human. First, we looked at the distribution of MHC-I peptide counts that were detected by MS across different mouse (Figure 3A) and human (Figure 3B) tissues. Expectedly, we noted that specific mouse organs yielded high numbers of MHC-I peptides (e.g., Spleen) whereas immune privilege organs (e.g., Brain and Testis) yielded low numbers of MHC-I peptides (Figure 3A). Very similar observations were made in humans (Figure 3B) (Marcu et al., 2021). In fact, direct comparison of MHC-I peptide counts between mouse and human tissues resulted in a positive correlation (R-squared value = 0.44) (Figure 3C). Next, we performed principal component analysis (PCA) of tissue dependent intensities of mouse and human MHC-I peptides (Figures 3D and 3E). PCA were performed from highly heterogeneous immunopeptidomic data integrating peptides and corresponding intensities presented by two mouse and 51 human MHC-I allotypes, respectively. Despite the high heterogeneity, our analysis revealed two main clusters in each species. Notably, immune-related organs clustered together in both species (see cluster one in Figures 3D and 3E). Immune clusters included Spleen, Bone Marrow, Lymph nodes, and Thymus (mouse), as well as other types of nonimmune related organs such as Kidney, Lung, Liver, and Colon. The described observations raised the following question: what are the MHC-I peptides that are either shared or unique across these tissues?

To address the above question, we created connectivity matrices, which summarize the number of MHC-I peptides shared and uniquely observed between all possible pairs of tissues in mouse (Figure 3F) and human (Figure S1B). The number of uniquely observed/tissue-specific peptides can be found along the diagonal of the connectivity matrices in Figure 3F and Figure S1B. In mice, we observed that 13% (961 out of the 7665 unique peptides found in mouse) of the total H2D^b^/K^b^ immunopeptidome was shared across Spleen, Bone Marrow, Kidney, Lung, Liver, and Colon (Figure 3F). As an example, 1881 peptides (25% of the total H2D^b^/K^b^ immunopeptidome) were shared between Spleen and Kidney, and 1381 peptides (18% of the total H2D^b^/K^b^ immunopeptidome) were shared between Bone marrow and Liver (Figure 3F). In humans, we observed that 4% of the total HLA-ABC immunopeptidome was shared across these six organs for all subjects. Once deconvolved by allotype or subject, we observed that, on average, 3% (range: 0.5% HLA-C∗07:04–9% HLA-A∗01:01 and B49:01) and 0.8% (range: 0.4% AUT-DN08–1.3%, AUT-DN12) of HLA-I peptides were shared across these organs, respectively (Figure S2). In contrast, larger fractions of MHC-I peptides were found to be uniquely observed in each species. Overall, 42% (3212 out of 7665 unique peptides) and 44% (32,187 out of 73,639 unique peptides) of the total H2D^b^/K^b^-immunopeptidome and HLA-ABC- immunopeptidome were uniquely observed in specific tissues, respectively. These peptides are further referred to as tissue-specific peptides. Thus, our data show, using the currently available technology, that a significant proportion of MHC-I peptides are tissue-specific whereas a relatively smaller proportion of peptides are shared across various immune and nonimmune organs, both in mouse and human (see also ‘limitations of the study’). To our knowledge, this is the first time that estimates of tissue-specific and shared MHC-I peptides are reported at the organism level. These two categories of MHC-I peptides may show distinct properties or trends, and were further investigated below.

### MHC-I peptides shared across multiple tissues are highly abundant and strong MHC-I binders

To investigate the properties of tissue-specific peptides versus those that are presented across a wide range of tissues, we sought to assess the influence of peptide abundance and MHC binding affinity on tissue distribution. Hence, we plotted the number of tissues in which a peptide has been detected against their average abundance or predicted MHC-I/HLA-I binding affinity (NetMHCpan4.0 rank score) (Figure S3 for mouse and Figures S4 and S5 for human). The human dataset has to be viewed in a subject-specific manner as each subject presents its own repertoire of HLA-I alleles. In mouse, we found that increasing cross-tissue presentation of MHC-I peptides strongly correlated with increasing peptide abundance and increasing affinity for the MHC-I molecules (decreasing NetMHCpan 4.0 rank score) (Figure S3). The same behavior was generally observed in humans, where peptides widely represented across tissues were highly abundant (Figure S4) and predicted to be strong HLA-I binders in all subjects (Figure S5). A possibility is that stable and abundant MHC-I peptides originating from abundant source proteins are easier to detect by MS. Beside this potential MS bias, our current data suggest that peptide abundance and binding affinity for MHC-I molecules are important properties that may contribute to the widespread or tissue-specific presentation of peptides in the mammalian MHC-I immunopeptidome.

### Tissue-specific MHC-I peptides arise from genes that are almost uniquely expressed in the peptide-producing tissue

**Figure 4:**
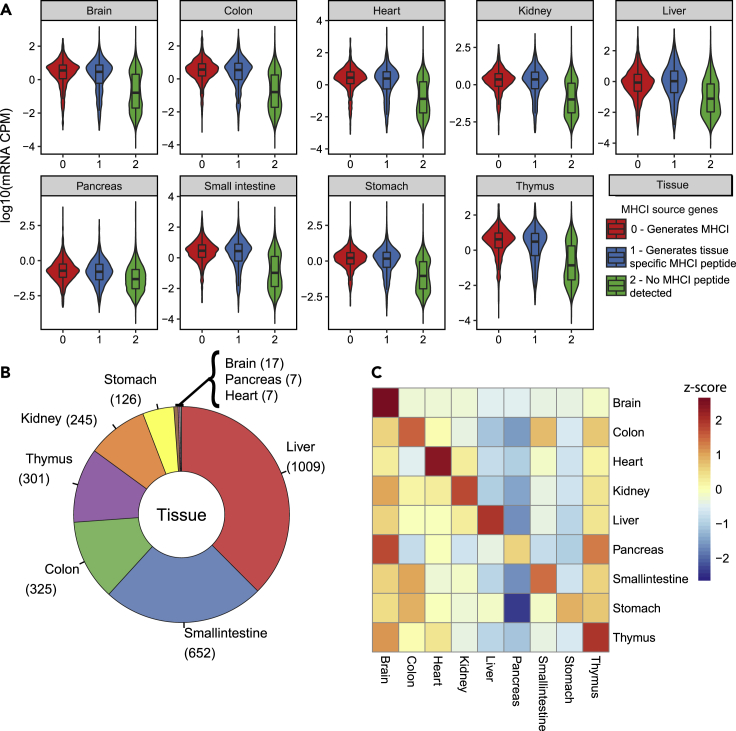
mRNA expression of MHCI source genes in multiple mouse organs (A) Violin plots depicting the distribution of mRNA expression of genes which generate MHCI peptides (0), genes which generate tissue specific MHC peptides (1) or does not generate MHCI peptides (2 B) Donut plot depicting the number of tissue-specific MHCI peptides found in tissues for which mRNA expression data is available (9 of 19 tissues sampled in the ‘B57BL/6 MHCI Ligand Atlas’. (C) Heatmap representing the average mRNA expression of genes coding for tissue-specific MHCI peptides across tissues. *Z* score is color coded

Expression of tissue-specific source proteins contributes to shaping the tissue-specificity of the human MHC-I immunopeptidome (Marcu et al., 2021). Pioneering work in mice also proposed that transcriptomic signatures of thymic cells can be conveyed to the cell surface in the MHC-I immunopeptidome (Fortier et al., 2008). Several MS-based immunopeptidomic studies have also shown that MHC-I peptides are preferentially encoded by genes that are actively transcribed, but all those studies were performed *in vitro* using cultured cell lines (Abelin et al., 2017; Fortier et al., 2008; Granados et al., 2012; Pearson et al., 2016). Hence, how gene expression shapes the composition of the mouse MHC-I immunopeptidome across many different tissues *in vivo* has never been reported to date. To address this, we first assigned every mouse MHC-I peptide found in the tissue draft atlas of the MHC-I immunopeptidome to its source gene. Using an RNA-Seq atlas of gene expression in mouse normal tissues (Söllner et al., 2017), we next assessed the transcript abundance of the MHC-I peptide source genes in nine tissues for which mRNA expression data were available (i.e., Brain, Colon, Heart, Kidney, Liver, Pancreas, Small intestine, Stomach, and Thymus) (Figure 4). From this RNA-Seq atlas, 94% of the identified MHC-I peptides were matched to mRNA entries. Then, using the matched dataset, we found that genes coding for any detectable MHC-I peptides as well as for tissue-specific MHC-I peptides were more actively transcribed compared to genes that were not coding for any detectable MHC-I peptides (Figures 4A and 4B).

Next, we reasoned that tissue-specific MHC-I peptides could derive from tissue-specific transcripts. To test this hypothesis, we averaged for every tissue the transcript abundance of genes coding for tissue-specific peptides and compared their expression across the nine tissues (Figure 4C). As depicted, we observed that brain-specific MHC-I peptides derived from genes that were uniquely expressed in the brain. Interestingly, liver-specific MHC-I peptides derived from genes that were predominantly, but not exclusively expressed in the liver—an expression pattern that was observed for seven out of nine tissues (colon, kidney, liver, heart, small intestine, stomach, and thymus; Figure 4C). Thus, we provide new evidence at the organism-scale that tissue-specific MHC-I peptides are generally encoded from genes that are highly expressed in the same tissue of origin. Together, these results are in accordance with conclusions drawn in humans (Marcu et al., 2021) and enforce the notion that gene expression plays a fundamental role in shaping the tissue specificity of the MHC-I immunopeptidome in mammals.

### MHC-I peptides that are broadly presented across many tissues are encoded by genes that are highly expressed and evolutionarily hyperconserved

**Figure 5:**
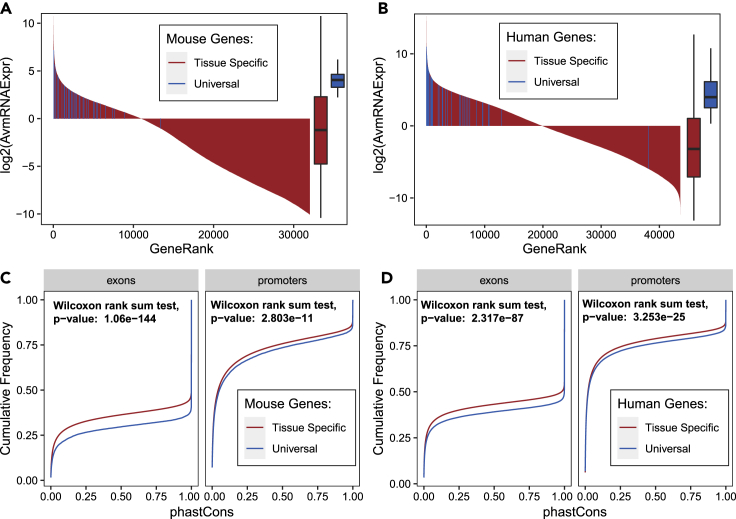
Expression and genetic conservation of genes coding for MHCI/HLAI peptides presented across most tissues (housekeeping/universal peptides) (A and B) mRNA expression of source genes of housekeeping/universal MHCI/HLAI peptides compared to all other mRNA transcripts in mouse (A) and human (B). (C and D) Exon and promoter conservation distributions of source genes of housekeeping/universal MHCI/HLAI peptides compared to source genes of tissue-specific MHCI peptides in mouse (C) and human (D).

Above, we provided evidence that the MHC-I immunopeptidome is composed of tissue-specific peptides as well as peptides that are widely presented across many different tissues. Although tissue-specific MHC-I peptides appear to stem from genes predominantly expressed in the original tissue, we asked whether MHC-I peptides that were presented across most tissues derived from highly transcribed genes across the entire human or mouse genome. To answer this question, we created a selection of MHC-I peptides that were widely represented among the sampled tissues, referred herein as ‘housekeeping/universal MHC-I peptides’ (Figure S6A). Although this selection is straightforward for the mouse data where we considered peptides identified in all 19 of the 19 tissues (35 selected peptides; 0.5%) as housekeeping/universal peptides, a more complex approach was needed to select those peptides in the human dataset where several subjects, each representing a specific set of HLA-I alleles, were present (827 peptides, 1.1%). Details about the selection of those peptides in the human immunopeptidome tissue draft are described in the methods section ‘Selection of Housekeeping/Universal Peptides’ and are visualized in Figures S6B–S6F and S7). First, we found that the selected MHC-I peptides originated from 38 to 251 source genes in mouse and human, respectively (Tables S1 and S2, and Figures S6 and S7). Importantly, we discovered that these genes were among the most transcriptionally expressed genes across the entire mouse (Figure 5A) and human (Figure 5B) genome. This result is in line with the above observation that widely presented peptides across the organism are of high abundance (Figures S3 and S4). Moreover, it is noteworthy that those housekeeping/universal MHC-I peptides did not preferentially originate from large (heavy) proteins, as it could have been expected because of the higher numbers of possible peptide antigen products from large proteins (Figure S8).

Genes expressed in the majority of tissues in an organism play vital functions, are evolutionarily hyperconserved and are widely referred to as housekeeping genes (She et al., 2009; Zeng et al., 2016; Zhu et al., 2008). Akin to housekeeping genes, peptides that are represented in most tissues across an entire organism—referred above to as housekeeping/universal MHC-I peptides—could also originate from hyperconserved proteins as they may have coevolved for millions of years with ancients and ubiquitous degradation systems to become the fundamental ground source of MHC-I peptides for most tissues. Hence, we hypothesized that universal MHC-I peptides are encoded by genes that are evolutionarily hyperconserved across evolution. To address this concept, we took advantage of the genome alignments between mouse and 59 vertebrates as well as between human and 99 vertebrates, made available by the UCSC Genome Browser (Lee et al., 2020) (see STAR Methods section ‘Conservation of source genes from universal MHC-I peptides’). To assess evolutionarily conservation across species, PhastCons scores (Siepel et al., 2005), which predict the probability of conservation for every base pair in the aligned genomes, were consulted for mouse and human genes of interest (see STAR Methods for details). When comparing the conservation scores of tissue-specific MHC-I peptide source genes with those from housekeeping/universal MHC-I peptide source genes, the latter were significantly more conserved at the Promoter and Exon level, both in Mouse (p value = 2.8 × 10^−11^; p value = 1.06 × 10^−144^) (Figure 5C) and Human (p value = 3.25 × 10^−25^; p value = 2.32 × 10^−87^) (Figure 5D). For example, the conservation probability (PhastCons score) of 70% of the more conserved Exons (Cumulative Frequency >0.3) of tissue-specific peptide source genes in mouse is greater than 20%, whereas the conservation probability of 70% of the more conserved Exons of housekeeping/universal peptide source genes in mouse is greater than 80%. Thus, this analysis indicates that tissue-specific versus housekeeping/universal MHC-I peptide source genes do not share the same degree of conservation across evolution. Together, our results suggest that highly expressed and hyperconserved genes contribute to the selected 0.5 and 1.1% of the mouse and human immunopeptidome that is shared across most tissues *in vivo*, respectively.

### Discovery of new components of the constitutive antigen processing and presentation network in mouse and human tissues

**Figure 6:**
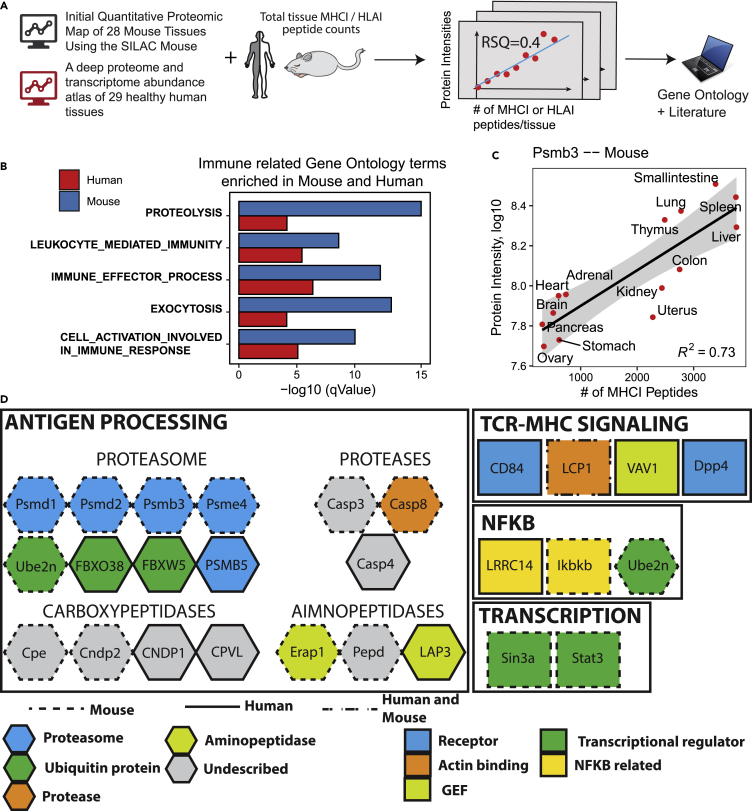
Correlation of protein abundances at the proteome-wide scale with the total number of MHCI or HLAI peptides detected across tissues (A) Protein expression data from protein expression maps of mouse and human tissues were correlated with the total number of MHCI or HLAI peptides detected per tissue. Correlations were simulated for every protein measured across nine or more tissues. Significantly correlating proteins were further investigated. (B) Gene Ontology terms enriched from 164 mouse and 120 human proteins whose abundance significantly correlates with the number of MHCI/HLAI peptides counted per tissue. (C) Example correlation of proteasome subunit Psmb3 in mice with MHCI peptides counted across tissues. (D) Protein modules identified from the global correlative analysis are associated with antigen generation, processing, and recognition. Mouse and human proteins annotated to enriched GO terms were manually curated from the literature and were classified based on their respective biological function: proteasome, aminopeptidase, carboxypeptidase, protease, ubiquitin protein, guanine nucleotide–exchange factor (GEF), actin binding protein and transcriptional regulator, and NFKB related. Proteins depicted in gray are uncharacterized enzymes of the antigen processing network.

Differential expression and activity of antigen processing and presentation proteins across tissues may contribute to the observed variability in the composition of the MHC-I immunopeptidome from one tissue to another (Rock et al., 2016). In this regard, transcript levels of HLA-I, TAP1/2, and immunoproteasome were very recently shown to correlate positively with the total number of MHC-I peptides detected across different human tissues (Marcu et al., 2021). To date, such correlative analysis has only been applied at the transcript level for a handful number of preselected immune-related genes and has never been performed at the protein level in a systematic fashion. Hence, we reasoned that an unbiased computational approach could be used to systematically identify any protein of the proteome for which their respective abundance across tissues correlates with the total number of MHC-I peptides across those same tissues. Therefore, we set out to apply this correlative approach at the proteome-wide scale using protein abundances measured across different mouse and human tissues from two tissue-based proteomics atlases generated by MS (Figure 6A) (Geiger et al., 2013; Wang et al., 2019).

First, we computed a total of 4,175 (on 4,175 gene coding proteins) and 70,656 (on 11,776 gene coding proteins) correlations in mouse and human, respectively (see STAR Methods). Importantly, we found a subset of 164 and 120 correlating proteins in mouse and human, respectively, whose abundance significantly correlated with the total number of MHCI peptide counts in a given tissue (p value < 0.01 and R-squared > 0.4 in Mouse; p value < 0.05 and R-squared > 0.4 for at least two subjects in Human) (Figures S9A and S9B, Tables S3 and S4). From the 164 mouse proteins, 122 correlated positively (74%) and 42 correlated negatively (26%) with MHCI peptide counts. Out of the 120 significantly correlating human proteins, 74 correlated positively (62%) and 46 negatively (38%) (Figure S10).

To broadly assess biological processes in which these proteins are implicated, we performed gene ontology (GO) analysis on these significantly correlating proteins (Table S5). From the top 50 most significantly enriched GO terms implicated in biological processes in mouse and human, 15 were shared across both species (Figure S11). Remarkably, the shared GO terms were attributed to proteins implicated in the regulation of the proteolysis, antigen processing, and immune response (Figures 6B and 6D). A prominent example protein, PSMB3 whose tissue dependent intensity very well correlates with the amount of MHCI peptides found in each tissue is shown in Figure 6C. Furthermore, manual curation of the literature allowed us to associate those proteins to specific functional modules known to orchestrate transcription (e.g., STAT3, NFKB), TCR-MHC signaling (e.g., LCP1, VAV1), and antigen processing (e.g., PSMB3/5, PSME4, LAP3, and ERAP1) (Figure 6D). Among the latter, many proteasome subunits, proteases, carboxypeptidases, and aminopeptidases were identified (Figure 6D). For example, PSMB3 is a component of the 20S core proteasome complex (Elenich et al., 1999; Huber et al., 2012); PSME4 is a proteasome activator subunit, also known as PA200 (Rêgo and Fonseca, 2019), and ERAP1 plays a central role in peptide trimming for the generation and presentation of MHC-I peptides (Serwold et al., 2002). For these three specific proteins, their abundance increased as a function of the number of MHC-I peptides (Figures 6C and S12 and Tables S3, S4, and S5). In contrast, we found the opposite trend for other proteins. For instance, abundance of Uchl1, Ube2n, and PSMB5 proteins decreased as a function of the number of MHC-I peptides (Figures S10B–S10D), the latter being known to be replaced by the immunoproteasome and thymoproteasome subunit PSMB8 and PSMB11 in immune and thymic cells, respectively (Murata et al., 2018). Most strikingly, we found four poorly characterized carboxypeptidases (CPE, CNDP1, CNDP2, and CPVL) showing significant correlations between protein abundance and number of MHC-I peptides across tissues (Figure 6D). This unexpected finding is interesting because very little is known about the role of carboxypeptidases in antigen processing. Therefore, furtherinvestigation is required to determine the precise role of CPE, CNDP1, CNDP2, and CPVL in shaping the global composition of the mammalian MHC-I immunopeptidome in health and diseases. Thus, our systems-level analysis allowed us to identify many known key players of the antigen processing network, thereby validating our computational approach, in addition to expanding the network through identification of new components. Collectively, our study provides an unprecedented source of information regarding the biogenesis of the mammalian MHC-I immunopeptidome and opens up new avenues to further explore the role of new proteolytic enzymes in antigen processing *in vivo*.

## Discussion

The components of the antigen processing and presentation pathway shape how T cells respond to self and nonself (Rock et al., 2016). Those components have been traditionally discovered using hypothesis-driven approaches or genomic screening of cell lines presenting a phenotype of interest (Burr et al., 2019; Neefjes et al., 2011; Paul et al., 2011). MS-based immunopeptidomic approaches have also been used to validate the impact of those proteins on the global composition of the MHC-I immunopeptidome using *in vitro* or *ex vivo* model systems (Alvarez-Navarro et al., 2015; Nagarajan et al., 2016; Verteuil et al., 2010). To date, no study has taken advantage of the uncharted combination of immunopeptidomic, proteomic, transcriptomic, and genomic data from a range of different primary tissues to infer the fundamental principles that form the mammalian MHC-I immunopeptidome. In fact, akin to systems immunology methods (Villani et al., 2018), we deployed in this study an unbiased immunopeptidomic data-driven strategy using multiple tissue-based omics datasets, both in mouse and human, to i) reinforce the notion that the composition of the mammalian MHC-I immunopeptidome is highly context-dependent, ii) provide fundamental information about the tissue-dependency, conservation, and biogenesis of the MHC-I immunopeptidome at the organism-wide scale, and iii) uncover new proteins that may collectively orchestrate the content and tissue-specificity of the MHC-I immunopeptidome.

In this study, we found that many proteins of the ubiquitin-proteasome degradation system as well as many proteases, aminopeptidases, and carboxypeptidases were more abundant in organs presenting a large number of MHC-I-peptide complexes. In addition, proteins known to negatively regulate protein degradation were found to be more abundant in organs presenting low numbers of MHC-I peptides. In fact, correlations between protein abundances and numbers of MHC-I peptides detected in tissues were found to be remarkably informative and could be used to systematically infer the role of new proteolytic enzymes in antigen processing. Proteolytic enzymes are critically important in antigen processing. Beside the proteasome, ∼20 proteases act in the MHC-I presentation pathway and can alter presented peptides (Lázaro et al., 2015). ERAP1 is probably the most relevant example here because this aminopeptidase plays a major role in antigen processing through N-terminal peptide trimming into the ER and is associated with a number of different autoimmune diseases (Hanson et al., 2018; Serwold et al., 2002). Other aminopeptidases such as leucine aminopeptidase 3 (LAP3) and peptidase D (PEPD) were showcased in this study. Most surprisingly, we identified four carboxypeptidases (CPE, CNDP1, CNDP2, and CPVL)—none of them reported so far to influence the repertoire of MHC-I peptides. These carboxypeptidases might represent new players of the antigen processing and presentation pathway. If tested and validated, such findings would be particularly fascinating because ACE (ACE) is the only ER-resident carboxypeptidase documented so far (Eiseniohr et al., 1992; Shen et al., 2008, 2011), and was shown to be immunologically relevant through production of minor histocompatibility antigens, polyoma virus epitopes, and HIV gp160 epitope (Neefjes et al., 2011). The use of chemical inhibitors and CRISPR technology, together with high-throughput immunopeptidomic experiments would be of great value in this context to systematically investigate the role of new proteolytic proteins in shaping the composition and heterogeneity of the MHC-I immunopeptidome in different cell and tissue types.

Two distinct categories of self-peptides were investigated in this study: those that are tissue-specific and those that are widely presented across most tissues, referred in this study as housekeeping/universal MHC-I peptides. Notably, our results show that these two categories of self-peptides share very distinct intrinsic features. The latter is composed of peptides that are highly abundant and strong MHC-I binders in addition to derive from highly expressed genes that are preferentially hyperconserved across evolution. In contrast, tissue-specific peptides are relatively less stable and are encoded by genes that are strongly expressed in the tissue of origin, but weakly or not expressed in most tissues. Such features may play a role in modulating T cell tolerance and autoimmune disorders. In fact, tolerance mechanisms through recognition of self-peptides, both in the thymus and in the periphery, are critical to eliminate or control self-reactive T cells that would otherwise lead to autoimmunity (Granados et al., 2015; Verteuil et al., 2012; Xing and Hogquist, 2012). In this regard, the current data show that a relatively large number of MHC-I peptides are tissue-specific, both in mouse and human. In theory, all those tissue-specific MHC-I peptides could be found in medullary thymic epithelial cells (mTEC). Presentation of tissue-specific MHC-I peptides in mTEC could be largely governed by autoimmune regulator (AIRE), the transcription factor that crucially regulates promiscuous gene expression for the establishment of self-tolerance (Anderson et al., 2002; Takahama et al., 2017). To date, the precise contribution of AIRE in shaping the repertoire of tissue-specific MHC-I peptides in mTEC remains undocumented, but deciphering its precise contribution, both qualitatively and quantitatively, would improve our understanding of T cell tolerance against tissue-specific self-MHC-I peptides. This is important because AIRE deficiency causes a failure in optimal promiscuous gene expression, and therefore in the establishment of self-tolerance in T cells, leading to the onset of autoimmune diseases in humans and mice (Takahama et al., 2017). Similarly, failure to self-tolerance against the other category of self-MHC-I peptides, those that are abundantly presented everywhere—i.e., the housekeeping/universal peptides—would have even more devastating consequences as self-reactive T cells would destroy all organs across the entire organism. Fortunately, we observed that genes coding for those housekeeping peptides are among the most expressed across entire genomes, hence, increasing the probability that those peptides will be abundantly presented in the thymus to trigger clonal deletion of immature self-reactive T cells recognizing those peptides. Moreover, we made the fundamental observation that housekeeping/universal peptides originate from hyperconserved genes. Therefore, the adaptive immune system may have evolved for 500 million years, a remarkable mechanism enabling the elimination of those T cells in a highly efficient manner. In contrast, controlling self-reactivity of T cells recognizing tissue-specific peptides might be more challenging, thereby rationalizing the need for peripheral tolerance processes to avoid tissue-specific autoimmunity (Matsumoto et al., 2019). Another causal logic to explain the occurrence of broadly presented MHC-I peptides to be hyperconserved would be the fact that the MHC molecules select for evolutionary conserved peptide sequences (binding motifs) resulting in the broad presentation of certain peptides with greatest conservation across evolution.

Another important observation in this study was that the multiple HLA-I allotypes expressed by a given individual may contribute unevenly to the composition of the MHC-I immunopeptidome from one organ to another. For instance, HLA-B40:02-associated peptides were found to be particularly enriched in the liver of a given individual compared to all the other organs. Overall, 37 enrichment patterns were observed across 27 specific HLA-I allotypes and 18 different tissues. This is an important basic information because peptide antigens that are processed and presented in a tissue-dependent fashion may cause differential phenotypic consequences in response to the same signal. For instance, in infectious diseases, *Plasmodium* parasites (malaria) and SARS-CoV-2 (COVID-19) have the ability to reach and infect many host tissues (Coban et al., 2018; Wadman et al., 2020). In this context, CD8^+^ T cells may behave very differently from one tissue to another following tissue-dependent processing and presentation of pathogen-derived peptide antigens, thereby likely impacting the overall efficiency of viral clearance by T cells. Interestingly, tapasin could play an important role in shaping the observed differential composition of the HLA-I immunopeptidome between allotypes and tissues, as it was recently proposed to expand the HLA-I peptide repertoire across humans, ultimately influencing immune responses to pathogens and vaccines (Bashirova et al., 2020). Moreover, tissue-dependent antigen presentation may lead to a web of tissue-resident memory T cells that functionally adapt to their environment to stop viral spread across the organism (Kadoki et al., 2017; Poon et al., 2021). Hence, tissue-specific variations in the MHC-I immunopeptidome likely play a role in controlling infections or determining the severity of a disease. One can anticipate that immunopeptidomics approaches will be increasingly powerful in the future to investigate the dynamics of the MHC class I antigen processing and presentation pathway *in vivo* and evaluate its impact on tissue-dependent T cell responses in the organism.

Systems understanding of MHC-I antigen presentation at the organism level is at an early stage. MS technologies are constantly evolving and we anticipate that the tissue-specificity of the MHC-I immunopeptidome will be further refined in the future. In fact, we envision that further improvement in proteomics and immunopeptidomics technologies will enable more robust, precise, and comprehensive measurements of proteomes and immunopeptidomes in healthy tissues as well as in response to a wide range of immunological perturbations. Integration of those measurements over time, together with new high-throughput TCR-MHC peptide interaction studies (Dendrou et al., 2018; Moritz et al., 2019), will help understand how widespread and tissue-specific changes in peptide processing and presentation impact tissue-dependent T cell responses, and hence, help understand interorgan communications between T cell networks to shape the organismal circuitry of immunity (Chevrier, 2019; Kadoki et al., 2017). From a synthetic biology perspective, in-depth understanding of how MHC-I-associated peptides are generated *in vivo* will enable accurate prediction of their dynamics, and ultimately, will accelerate the engineering of new biological systems to control their presentation and function in immunity.

### Limitations of the study

This bioinformatic study characterizes the *in vivo* immunopeptidome of mouse and human. This is the first study of that kind and is critical for understanding constitutive antigen presentation. However, the study has a number of technical limitations, which would need to be considered for the design of rigorous follow up studies: 1) Bulk tissues were used. Therefore, the contribution of various stromal cells vs resident bone marrow-derived cells was not considered. There are also limitations in analyzing thymus as a whole organ comprised of negative and positively selecting cells as well as technical limitations in false negative peptide detection. 2) Immunopeptidome analysis using the currently available protocols is highly biased to peptides that bind MHC molecules with high affinity, although low affinity peptides can be immunogenic (Yewdell and Bennink, 1999). 3) MS methods have bias for detecting peptide ligands from different HLA allotypes, with different charge properties and consensus motifs (Demmers et al., 2019). For this reason, quantitative comparisons within the same patient (same allele) are interpretable, but cross-comparison between individuals of different HLA allotypes is difficult to interpret in terms of immunopeptidome coverage, and especially peptide intensity. 4) The sensitivity of the currently available LC-MS technology is limited. Therefore, precise number/fraction of tissue-specific peptides reported in this study will likely change as the technology evolves. 5) Related to point (4), tissue data has not been normalized, meaning that less material and MHC expression levels in different tissues will guide the overall sequencing depth, and therefore define the overlap of presented peptide sequences between the tissues. 6) Biological replicates to assess reproducibility of peptide recovery from each organ of different mice have not been performed in (Schuster et al., 2018). On that note, multiple replicates could be performed in future mouse and human immunopeptidomic mapping efforts to show statistical significance across tissues. 7) The bias of LC-MS acquisition toward the most abundant peptide species may define the relationship with RNA transcript abundance. 8) LC-MS database interpretation could lead to a bias of identifying peptides from non-variable regions because spectral interpretation did not include accurately matched personalized databases. 9) Related to point (8), the mRNA database used did not include noncanonical mRNA. Therefore, many noncanonical MHC-I peptides were not identified but could be identified in future studies by performing RibSeq on the same samples, as described (Chong et al., 2020; Cuevas et al., 2021). 10) The conclusion that 'hyperconserved' regions are preferentially presented need very careful further validation. 11) Another limitation is the inability of the NetMHCpan suite tools to correctly annotate peptides to HLA alleles that are less characterized. Hence, further development and application of new peptide clustering and HLA peptide binding algorithms are expected to improve the accuracy of peptide annotation in future immunopeptidomic mapping efforts.

## STAR★Methods

### Key resources table

**Table:** 

REAGENT or RESOURCE	SOURCE	IDENTIFIER
**Deposited data**		

Mouse immunopeptidomics	Schuster et al. (2018)	https://doi.org/10.1038/sdata.2018.157
Human immunopeptidomics	Marcu et al. (2021)	https://doi.org/10.1136/jitc-2020-002071
Mouse proteomics	Geiger et al. (2013). doi: https://doi.org/10.1074/mcp.M112.024919. Epub 2013 Feb 22. PMID: 23436904; PMCID: PMC3675825	https://doi.org/10.1074/mcp.m112.024919
Mouse transcriptomics	Söllner et al. (2017)	https://doi.org/10.1038/sdata.2017.185
Human transcriptomics (accessed January 10^th^ 2020), the dataset used was: ‘GTEx_Analysis_2017-06-05_v8_RNASeQCv1.1.9_ gene_median_tpm.gct’	GTEX repository https://www.gtexportal.org/home/(accessed January 10^th^ 2020)	GTEx_Analysis_2017-06-05_v8_RNASeQCv1.1.9_ gene_median_tpm.gct
Human proteomics	Wang et al. (2019). doi: https://doi.org/10.15252/msb.20188503. PMID: 30777892; PMCID: PMC6379049	https://doi.org/10.15252/msb.20188503
PhastCons Gene conservation data (Human)	http://hgdownload.soe.ucsc.edu/goldenPath/hg38/multiz100way/(accessed June 5^th^ 2020)	DOI:https://doi.org/10.18129/B9.bioc.TxDb.Hsapiens.UCSC.hg38.knownGene
PhastCons Gene conservation data (Mouse)	http://hgdownload.soe.ucsc.edu/goldenPath/mm10/multiz60way/(accessed June 5^th^ 2020)	DOI: https://doi.org/10.18129/B9.bioc.TxDb.Mmusculus.UCSC.mm10.knownGene

**Software and algorithms**		

R	https://www.r-project.org/	
R Studio	https://www.rstudio.com/	
MHC-I Atlas	In-house code	https://github.com/CaronLab/MHCIatlas

### Resource availability

#### Lead contact

Further information and requests should be directed to the lead contact, Dr. Etienne Caron (etienne.caron@umontreal.ca)

#### Materials availability

This study did not generate new unique reagents.

### Experimental model and subject details

Six datasets from previously reported studies were used (see each original study for details): 1) a mouse immunopeptidomic dataset generated from 19 primary tissues (male and female C57BL/6) (Schuster et al., 2018), 2) a human immunopeptidomic dataset generated from 29 primary tissues and 21 different individuals (Marcu et al., 2021), 3) a mouse proteomic dataset generated from 28 primary tissues (Geiger et al., 2013), 4) a human proteomic dataset generated from 29 primary tissues (Wang et al., 2019), 5) a mouse transcriptomic dataset generated from 13 normal tissues (male C57BL/6) (Söllner et al., 2017), and 6) a human transcriptomic dataset from the GTEX repository (https://www.gtexportal.org/home/; see STAR Methods details below).

### Method details

#### Retrieval and preparation of omics data from the literature

##### Mouse immunopeptidome

Raw data from the mouse immunopeptidome dataset (Schuster et al., 2018) were downloaded and re-analyzed using “PEAKS 9 (Bioinformatics Solutions Inc., Waterloo, Ontario, Canada)” (Tran et al., 2018). For the analysis, a Peaks 9 project was generated for each pulldown experiment and parameters for the peptide searches used were non-specific digestion (Enzyme = None), the instrument was set as ‘Orbitrap’, Fragmentation as ‘HCD’ and ‘DDA’ was used for the acquisition method specified. Furthermore, we set the precursor mass to 10ppm (monoisotopic mass), fragmentation to 0.01Da and we included Oxidation(M) and Deamidation (NQ) as post translational modifications (PTM’s). We selected the option ‘Estimate FDR with decoy-fusion’ to assess false discovery rates. From these results, peptides identified with an FDR<5% were exported and further assessed for binding to the MHC-I alleles H2Kb and H2Db using NetMHCpan4.0 (Jurtz et al., 2017). Peptides with a length of 8,9,10,11 or 12 amino acids and a NetMHCpan-4.0 Rank score smaller than 2.0 (Rank ≤ 2.0) were selected as MHC-I peptides. A collection of all mouse MHC-I peptides is made available in Table S1. All downstream data analysis is based on this set of MHC-I peptides.

##### Mouse RNAseq data

Mouse RNAseq data were obtained from (Söllner et al., 2017) supplemental information and can be found in Table S1. Data were used for further analysis in the form provided.

##### Mouse proteomics data

Mouse proteomic data were downloaded from (Geiger et al., 2013) supplemental information and can be found in Table S3. Protein intensities presented in Table S3 are the reported label free intensities normalized across tissues.

##### Human immunopeptidome

Human immunopeptidome data were obtained from Marcu et al. (2021) and represent the 2020.06 release of the dataset as can be accessed at https://hla-ligand-atlas.org/. Mass spectrometry data acquisition and subsequent database searches are described in the method sections ‘Mass spectrometric data acquisition’ and ‘Database search with MHCquant’ in Marcu et al. (2021), respectively. Please refer to to these two method sections to access all the details and parameters regarding data acquisition and database searches for HLA class I peptide identification. Next, peptides from this dataset were predicted for HLA-I binding using NetMHCpan4.0 (Jurtz et al., 2017). Only alleles present in the donor database [based on allele genotyping as described in Marcu et al.] were predicted. Out of six alleles genotyped to each donor, the allele with the lowest/best NetMHCpan-4.0 Rank score was assigned to a given peptide. For the analysis presented in this manuscript, peptides with a NetMHCpan-4.0 rank smaller or equal than 2 (Rank<=2) were considered HLA-I peptides. The quantitative information, as reported by MHCquant (Bichmann et al., 2019), was also used in the current manuscript. Raw peptide intensities were used as approximative quantitative information and no normalization was performed due to the heterogeneous nature of pulldowns and primary tissue samples. A complete list of peptides including metadata can be found in Table S2. In Marcu et al. (2021), all HLA-I peptides identified were compared to peptides found in the IEDB and the SysteMHC Atlas, and a selected subset of cryptic peptides were validated using synthetic isotope-labeled peptides (Marcu et al., 2021). *In vitro* HLA-peptide binding assays (Sidney et al., 2013) will be performed in the future for cryptic peptides as well as for canonical peptides that are predicted to bind several HLA-I allotypes.

##### Human RNAseq data

Human RNAseq data were obtained from the GTEX repository https://www.gtexportal.org/home/(accessed January 10th 2020), the dataset used was: ‘GTEx_Analysis_2017-06-05_v8_RNASeQCv1.1.9_gene_median_tpm.gct’. The subset of data used for this manuscript can be found in Table S2. Data were used for further analysis in the form provided.

##### Human proteomics data

Human proteomics data were obtained from (Wang et al., 2019). The subset of data used for this publication can be found in Table S4. Data were used for further analysis in the form provided, unless stated differently.

#### Principal component analysis of immunopeptidome data

Principal component analysis and visualization was performed in R using the FactoMineR package (Lê et al., 2008). Input variables consist of 19 mouse tissues and 28 human tissues for which immunopeptidomic data are available (note that human thymus tissues were sampled differently and were excluded from this analysis). For each tissue, a vector of individual peptide intensities (log10 transformed) was loaded. The first two dimensions accounting for most of the variability in the data were plotted (Figures 2D and 2E).

#### Tissue connectivity maps

For every possible pair of tissues, the number of overlapping peptides was determined for the mouse and human immunopeptidomes, respectively. A peptide was considered overlapping if an intensity value had been reported in both tissues. A connectivity matrix was generated from the resulting data for mouse and human, respectively (Figures 2F and S1B). Noteworthily, the number of peptides unique to a given tissue is depicted along the diagonal of depicted heatmaps.

#### Tissue-dependent representation of HLA alleles

The proportion of peptides represented by a specific allele in a given tissue was calculated for every subject. Similarly, the mean proportion of every allele across tissues was calculated for every subject. These values were then used to calculate the over- or under-representation of each allele in a tissue compared to the mean as follows:

Subject dependent allele enrichment in tissues:

$$\frac{\%\left[Allele\right]in\;Tissue\;k}{\left(\sum_{k=1}^{n}\left[Allele\right]in\;Tissue\;k\right)/n}=Tissue\;dependent\;allele\;enrichment$$

Examples for subject specific allele representations can be found in Figures 2B–2D. In order to assess trends across all subjects, we calculated the mean of these over- and under-representation values for all alleles across all subjects. To find trends among the data, we focused only on alleles over-represented by, on average, at least 1.5-fold in a given tissue across all subjects. Results are depicted in Figure 2E.

#### Connecting mouse immunopeptidomic data with mouse RNAseq data

Source genes of mouse MHC-I peptides available from the Peaks results were mapped to ENSEMBL identifiers using the mouse annotation package org.Mm.eg.db in R (https://doi.org/10.18129/B9.bioc.org.Mm.eg.db). These source genes were then mapped to the genes in the RNAseq dataset (Söllner et al., 2017) to assess their tissue-dependent RNAseq expression (Table S1). All mappings between different gene identifiers were performed using the R package AnnotationHub (https://doi.org/10.18129/B9.bioc.AnnotationHub).

#### Source genes from tissue-specific MHC-I peptides in mouse (Mouse source genes)

Genes mapped to a peptide which is present in only one of the nineteen tissues analyzed in the mouse immunopeptidome are considered to be source genes of tissue specific MHC-I peptides. We have not assessed to what extend additional MHC-I peptides from such a gene are represented across tissues (for genes where more than one MHC-I peptide was identified). We found 2448 source genes from tissue-specific MHC-I peptides in mouse.

#### Tissue dependent expression of mouse source genes

In order to visualize the expression of source genes originating from tissue-specific MHC-I peptides, we mapped the 2448 source genes we found in mouse to the mRNA expression atlas published by (Söllner et al., 2017). For the 9 tissues where transcriptomics and proteomics data were available, we extracted mRNA expression levels of the mouse source genes. Expression levels were then averaged across each tissue, grouped by the source tissue (Tissue in which gene represents immunopeptides). Within each source tissue group, a z-score for the average expression value of genes was calculated. The resulting matrix is visualized in Figure 4C.

Note: Z-scores were calculated as follows:

$$z-score=\frac{Source\;gene\;expression\;in\;given\;tissue\;-\;Mean\;source\;gene\;expression\;across\;tissues}{Standard\;deviation\;of\;source\;gene\;expression}$$

#### Conservation of source genes from universal MHC-I peptides (Mouse)

Universal MHC-I peptides are defined as MHC-I peptides present in all of the 19 sampled mouse tissues (Figure S6A). Peptides from 38 genes were found in the mouse dataset (Table S1). We calculated the conservation of the exon and promoter regions of the corresponding source genes and compared their genetic conservation to those from source genes of tissue specific MHC-I peptides. Conservation scores were extracted in form of PhastCons conservation probabilities (Siepel et al., 2005; Siepel and Haussler, 2004) from publicly available multiple alignments of the mouse genome and the genomes of 59 vertebrates (http://hgdownload.soe.ucsc.edu/goldenPath/mm10/multiz60way/) from the UCSC genome browser (https://genome.ucsc.edu/index.html) (accessed June 5th 2020). BigWig files containing PhastCons scores for the mouse genome were downloaded and queried for the genes of interest using the R package rtracklayer (Lawrence et al., 2009) together with gene positional information from the ‘TxDb.Mmusculus.UCSC.mm10.knownGene’ database provided by the UCSC genome browser (https://doi.org/10.18129/B9.bioc.TxDb.Mmusculus.UCSC.mm10.knownGene). PhastCons scores for nucleotides of the exon and promoter regions of housekeeping and tissue-specific source genes were extracted from the BigWig files. Promoter regions were defined as 200 bases downstream and 2000 bases upstream of the transcription start site. Conservation scores were then calculated using a 12-base pair sliding window along the extracted genetic regions and the maximum PhastCons value was used as the conservation score. The cumulative frequency of these PhastCons values for the exon and promoter regions of source genes from universal MHC-I peptides and source genes from tissue-specific MHC-I peptides were calculated and compared using the Wilcoxon rank sum test, respectively. This analysis and workflow were inspired by Zeng et al. (2016) and Zhu et al. (2008) who investigated the genomic conservation of housekeeping genes compared to tissue specific genes in mouse and human, respectively. Furthermore, ideas for the implication of PhastCons conservation rates were derived from Sun et al. (2014).

#### Annotating the molecular weight of MHC-I peptide source genes (Mouse)

Molecular weights of proteins were retrieved from www.uniprot.org (Complete *Mus musculus* proteome, reviewed + un-reviewed proteins, accessed June 17 2020). Uniprot identifiers were matched to ENSEMBL gene identifiers and used for analysis.

#### Connecting human immunopeptidomic data with human RNAseq data

Source genes of human MHC-I peptides were mapped to ENSEMBL identifiers using the human annotation package org.Hs.eg.db in R (org.Hs.eg.db: Genome wide annotation for Human. R package version 3.8.2). These source genes were then mapped to the genes in the RNAseq dataset (‘GTEx_Analysis_2017-06-05_v8_RNASeQCv1.1.9_gene_median_tpm.gct’) to assess their tissue-dependent RNAseq expression (Table S2). All mappings between different gene identifiers were performed using the R package AnnotationHub (https://doi.org/10.18129/B9.bioc.AnnotationHub).

#### Source genes from tissue-specific MHC-I peptides (Human)

Source genes representing one or more MHC-I peptides that were measured in only one tissue sample in the human immunopeptidome dataset were considered source genes from tissue-specific MHC-I peptides. In human we found 12,095 of such genes. Similar to the mouse analysis, we did not assess to what extendt these genes yield additional peptides present in more than one tissue sample.

#### Conservation of source genes from universal MHC-I peptides (Human)

Defining source genes from universal MHC-I peptides in human is less straightforward compared to the mouse due to the heterogeneity of subjects from which tissues were sampled and HLA alleles representation. Hence, we defined a source gene from universal MHC-I peptides in the available human immunopeptidome as a gene for which one or more MHC-I peptides were either 1) present across all tissues in at least two patients or 2) present across all samples in which the assigned HLA allele was present or 3) among the top 100 peptides identified the most frequently across all measured samples, independent of allele or subject (Figures S6B–S6F). In order to avoid a bias towars peptides from donors where only few tissues were sampled, we focused only on donors where 14 or more tissues were sampled. This analysis resulted in a total of 251 source genes from universal MHC-I peptides (Figure S7 and Table S2).

Conservation analysis was performed using PhastCons retrieved from an alignment of the hg38 human genome with 99 vertebrates. Data were downloaded from the UCSC genome browser at http://hgdownload.soe.ucsc.edu/goldenPath/hg38/multiz100way/(accessed June 5th 2020). Genetic positions of genes of interest (genes from universal and tissue-specific MHC-I peptides) were mapped using the ‘TxDb.Hsapiens.UCSC.hg38.knownGene’ database (https://doi.org/10.18129/B9.bioc.TxDb.Hsapiens.UCSC.hg38.knownGene) and conservation scores were calculated and compared the same way as the mouse conservation scores.

#### Annotating the molecular weight of MHC-I peptide source genes (Human)

Molecular weights of proteins were retrieved from www.uniprot.org (Complete *Homo sapiens* proteome, reviewed + un-reviewed proteins, accessed June 17 2020). Uniprot identifiers were matched to ENSEMBL gene identifiers and used for analysis.

#### Computing and analyzing protein wise correlation between tissue MHC-I peptide counts and protein abundances in mouse and human

Protein expression data from mouse and human proteomic tissue drafts (Tables S3 and S4) were obtained from (Geiger et al., 2013) for mouse and (Wang et al., 2019) for human. Both datasets were chosen due to their recency and wide range of tissues sampled. Correlations between the expression pattern of a given protein across tissues and the overall number of MHC-I peptides sampled across tissues in mouse or human subjects were measured. Expression values (log10 transformed) of each protein across tissues were plotted against the number of total MHC-I peptides identified in each tissue and R-squared and p values were computed if more than 9 measurement pairs (expression value and total number of MHC-I peptides) were available. The R package ‘stats’ and the implemented function ‘lm’ was used to generate linear fits and R-squared values. For the human dataset, correlations were calculated for immunopeptidome data from every subject where above criteria were fulfilled. Expression values from jejunum and duodenum from the human proteomics dataset (Wang et al., 2019) were averaged and paired with total MHC-I peptide counts in the small intestine. p-values and R-squared values were reported. For the human data, we required p values < 0.05 and R-squared values > 0.4 in at least two patients to consider a correlation to be non-random (Figure S9A). For the mouse data where only one donor is available, correlations with p values < 0.01 and R-squared > 0.4 were considered to be non-random observations (Figure S9B). Correlation data for all proteins of the mouse and human datasets can be found in Tables S3 and S4, respectively.

#### Functional proteomic analysis

Gene set enrichment analysis (GSEA; http://www.broad.mit.edu/gsea/) was performed using GSEA software and the Molecular Signature Database (MsigDB) on proteins from systematic cross-tissue analysis of MHC class I peptides and protein expression. Top 50 significant gene sets using the Gene Ontology modules overlap analysis were considered significant with p value and FDR <0.05. We acknowledge our use of the GSEA, GSEA software, and MSigDB (Subramanian et al., 2005). Results can be found in Table S5.

### Quantification and statistical analysis

#### Significance of the correlations between tissue MHC-I peptide counts and protein abundances in mouse and human

As a first step, the significance of the correlations was defined based on a previous proteomics study (Kubiniok et al., 2017). In brief, Kubiniok et al. showed that R-squared values > 0.4 generally imply less than 1% false positive among fitted data compared to a randomized dataset of the same nature [see Figure S2 in Kubiniok et al. (2017) for details]. Then, we used a similar approach in the current study to assess the behavior of the dataset and found that the number of false positives is generally less than 5% for R-squared values above 0.4 (Figure S9A). Based on these results, we decided to choose an R-squared value of 0.4 as a general measure for a cut-off value together with the p value cut-off for this type of proteomics data. Accordingly, we show in Figure S9B and S9C that the R-squared value cut-off of 0.4 adds to the stringency of data that were selected when using a p value of 0.05 (Human) or 0.01 (Mouse) as a cut-off. Next, we used the R package stats and the function ‘lm’ (https://www.rdocumentation.org/packages/stats/versions/3.6.2/topics/lm) to apply the statistical method and generate the fitted curves. Note that we used only proteins for which at least ten measurements were available since ten data points were shown to be the minimal requirement for linear regression, as previously reported (Jenkins and Quintana-Ascencio, 2020). Hence, we systematically excluded proteins with less than ten measurements from the analysis. Apart from that, no immunopeptidome data was excluded from the mouse and human dataset described. Furthermore, we validated our method by crosschecking for an enrichment of true positive hits amongst the selected data. For example, prominent proteins involved in the degradation of proteins and generation of MHC-I peptides, like Psmd1, Psme4 and Erap1, show correlation values close to the set R-squared value cut-off (Figure S12).

## Data Availability

• Source data statement. This paper analyzes existing, publicly available data. These accession numbers for the datasets are listed in the key resources table. • Code Statement. All original code has been deposited at https://github.com/CaronLab/MHCIatlas and is publicly available as of the date of publication. DOIs are listed in the key resources table. Download of the source code can be performed directly from the above link. Alternatively, the package can be installed in R using the ‘install_github’ function from the ‘devtools’ package as shown below: > devtools::install_github('CaronLab/MHCIatlas') The ‘MHCIatlas’ R package includes 30 functions to reproduce the data-analysis presented in this manuscript as well as the immunopeptidomic, proteomics and transcriptomics datasets used. A quick guide to install and use the R package ‘MHCIatlas’ is provided as supplemental information in form of the ‘MHCIatlas user guide’ pdf document (Data S1).• Any additional information required to reanalyze the data reported in this paper is available from the lead contact upon request.

## Supplemental information

Document S1. Figures S1–S12 and Data S1

Table S1. Mouse immunopeptidome and transcriptome, related to Figures 4 and 5

Table S2. Human immunopeptidome and transcriptome, related to Figure 5

Table S3. Mouse proteome, related to Figure 6

Table S4. Human proteome, related to Figure 6

Table S5. GeneOntology enrichments of correlating mouse and human proteins, related to Figure 6

## Footnotes

Supplemental information can be found online at https://doi.org/10.1016/j.isci.2022.103768.
