# Supplementary material for: Understanding the constitutive presentation of MHC class I immunopeptidomes in primary tissues
Source: iScience. 2022 Jan 18;25(2):103768. doi: 10.1016/j.isci.2022.103768 (PMC8810409; doi:10.1016/j.isci.2022.103768)
Supplement: Document S1. Figures S1–S12 and Data S1 [file mmc1.pdf]

iScience, Volume *25*

Supplemental information

Understanding the constitutive presentation of MHC class I immunopeptidomes in primary tissues

Peter Kubiniok, Ana Marcu, Leon Bichmann, Leon Kuchenbecker, Heiko Schuster, David J. Hamelin, Jérôme D. Duquette, Kevin A. Kovalchik, Laura Wessling, Oliver Kohlbacher, Hans-Georg Rammensee, Marian C. Neidert, Isabelle Sirois, and Etienne Caron

Supplemental Information

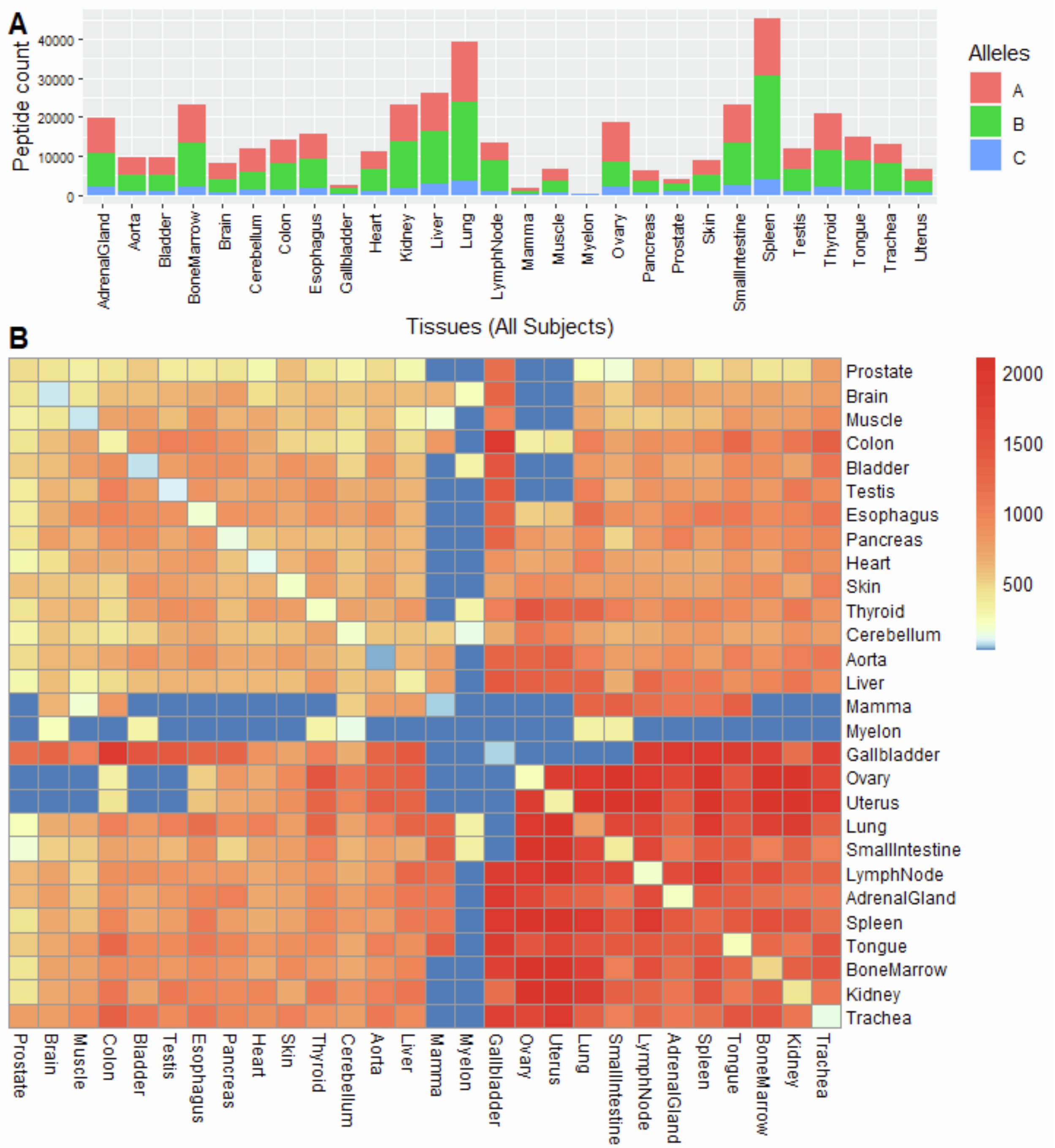

Figure S1. Human immunopeptidome, Related to Figure 2 and Figure 3. (A) Distribution of HLAI peptide alleles across tissues in the human immunopeptidome. **(B)**This human heat map integrates immunopeptidomic data from 38 HLAI allotypes and 13 different subjects. The human heat map is not deconvoluted by HLA allotype nor subject and therefore provide a bird’s eye view of the human class I immunopeptidome. Note: The number of uniquely observed/tissue-specific peptides can be found along the diagonal.

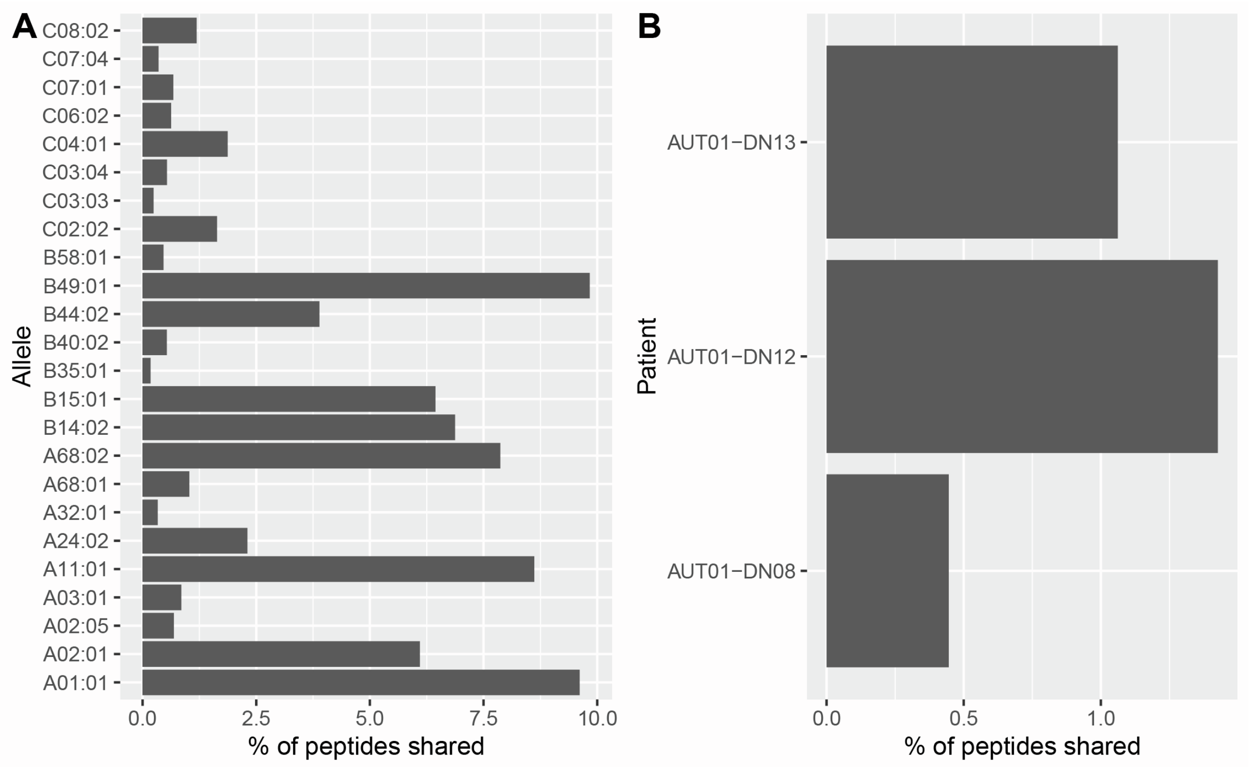

Figure S2. Proportion of peptides shared across Colon, Spleen, Liver, Lung, Bone marrow and Kidney, Related to Figure 3. (A) Deconvoluted by best allele for which all 6 tissues were sampled. **(B)** Deconvoluted by subjects for which all six tissues were sampled.

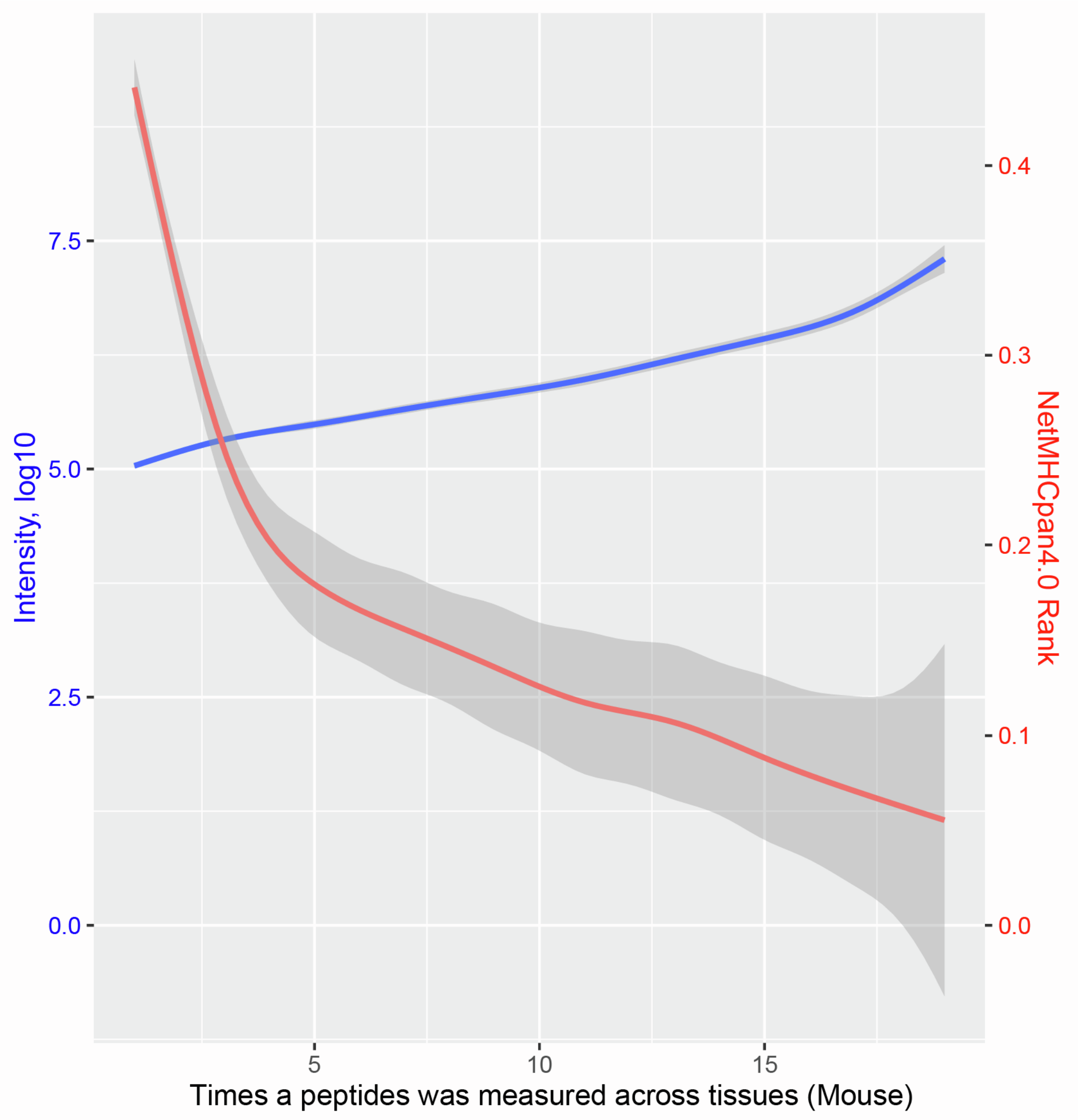

Figure S3. NetMHCpan4.0 rank and MHCI peptide intensities plotted against the number of measurements across tissues in the mouse dataset, Related to Figure 4 and Figure 5.

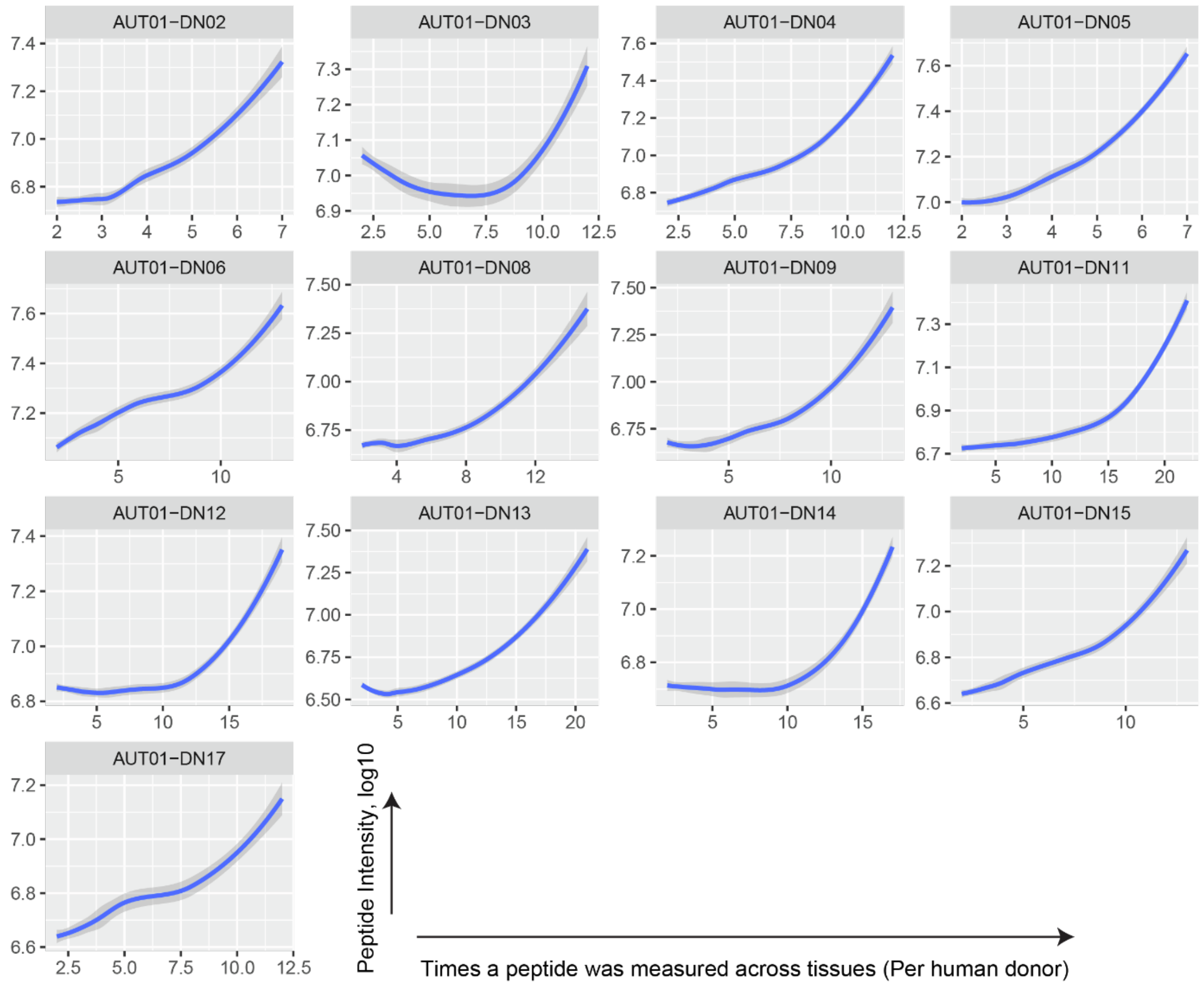

Figure S4. MHC I peptide intensities plotted against the number of measurements across tissues in the human dataset, Related to Figure 4 and 5. Each panel represents one subject.

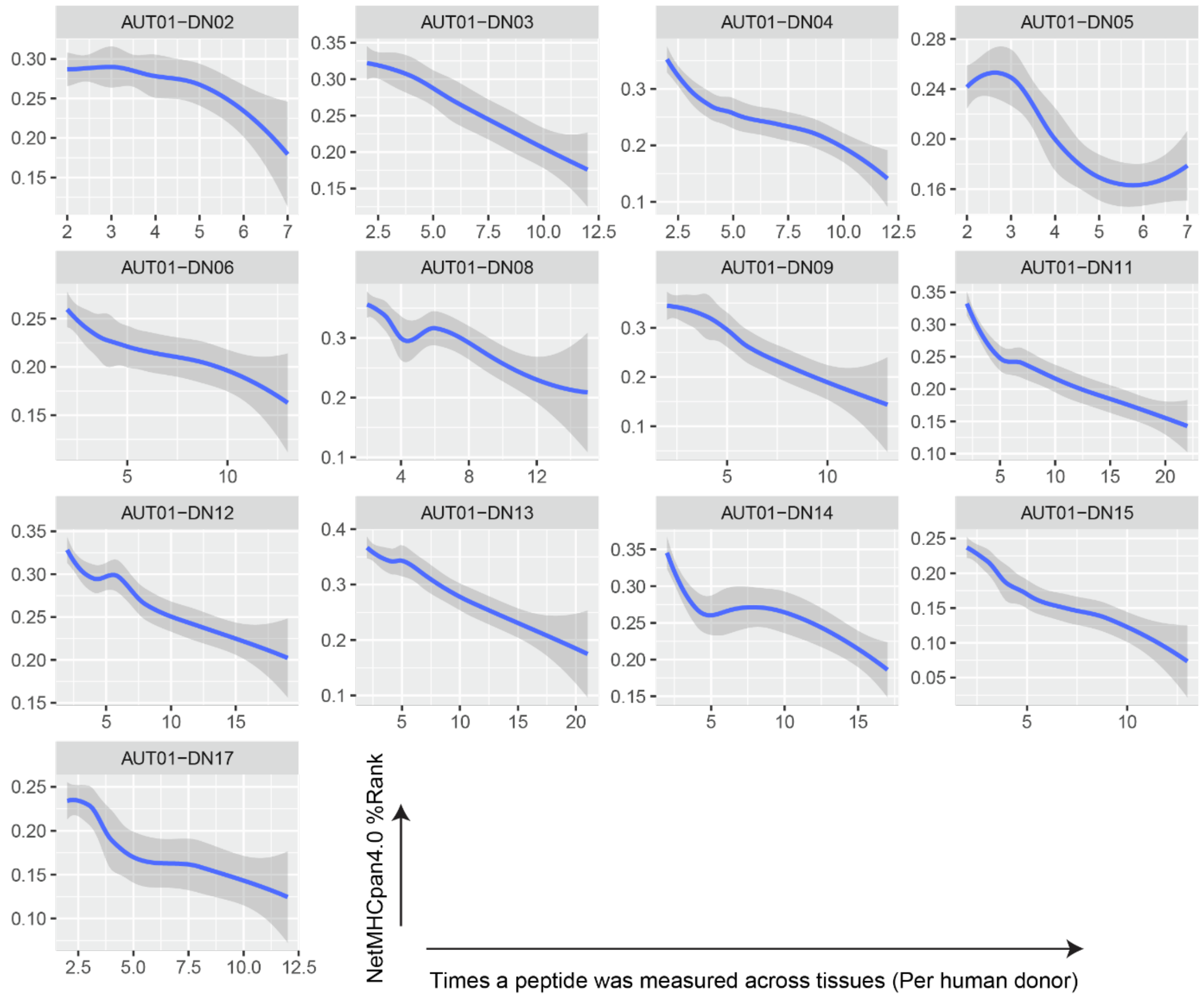

Figure S5. NetMHCpan4.0 rank (best allele) plotted against the number of measurements across tissues in the human dataset, Related to Figure 4 and Figure 5. Each panel represents one subject.

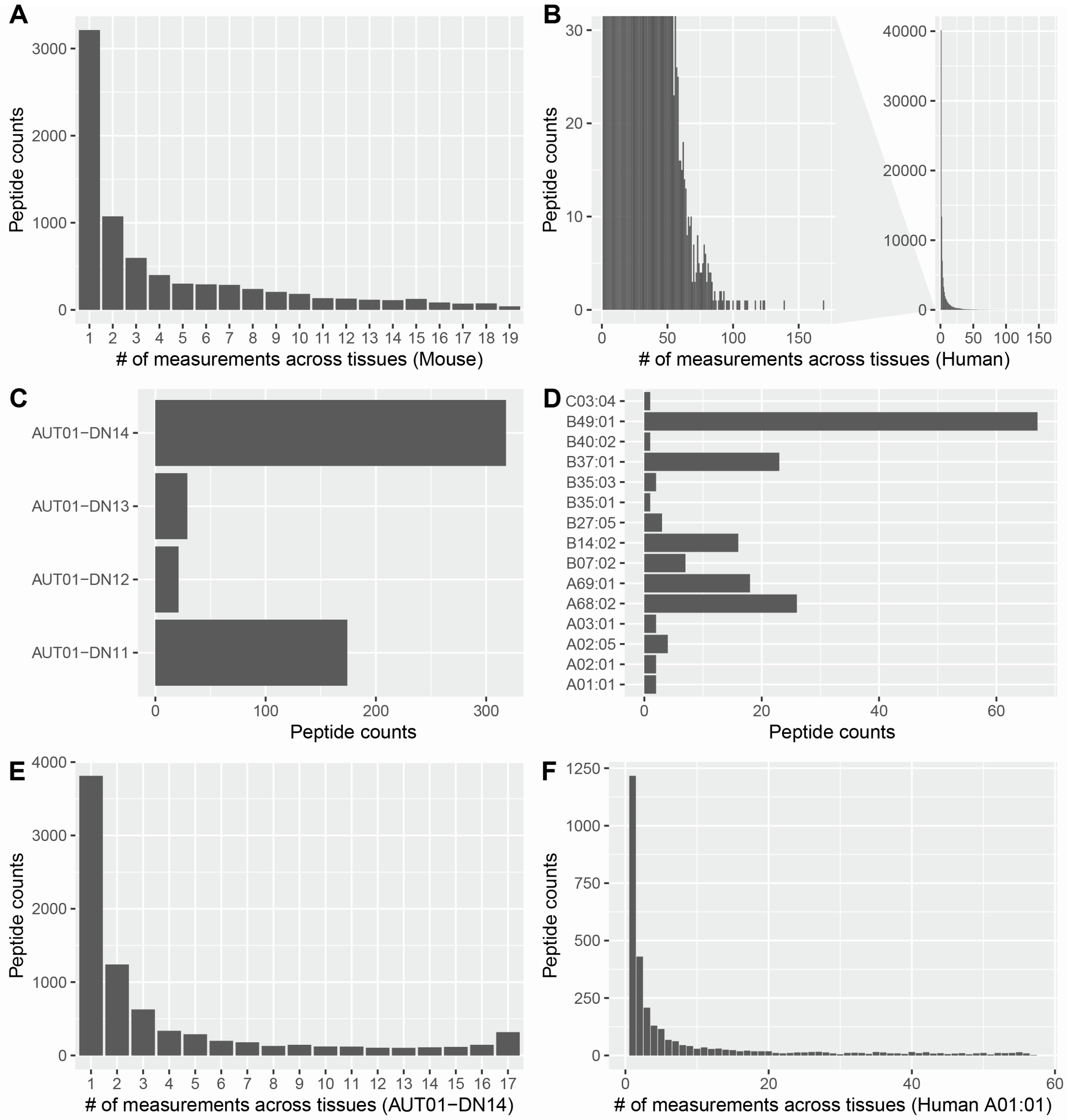

Figure S6. Identification of the mouse and human tissue-independent MHCI peptides and their corresponding source genes, Related to Figure 4 and Figure 5. (A) Histogram showing the number of MHCI peptides presented in one or more tissues. 1 measurement are MHCI peptides identified in only one tissue whereas 19 measurements are MHCI peptides identified in all the 19 tissues. Mouse source genes coding for MHCI peptides that were measured across more than 17 tissues were considered for further analysis. **(B)** Source genes of the top 100 peptides from the human dataset measured the most frequently across tissues were considered 'source genes of universal peptides' . **(C)** In addition to B, source genes that present peptides across all tissues in at least two patients are also considered as 'source genes of universal peptides'. **(D)** In addition to B and C, source genes that present peptides across all tissues for at least one allele are considered 'source genes of universal peptides'. **(E)** Example of the distribution of peptide counts across tissues in AUT-DN14. **(F)** Example distribution of peptide counts across all samples containing allele A01:01.

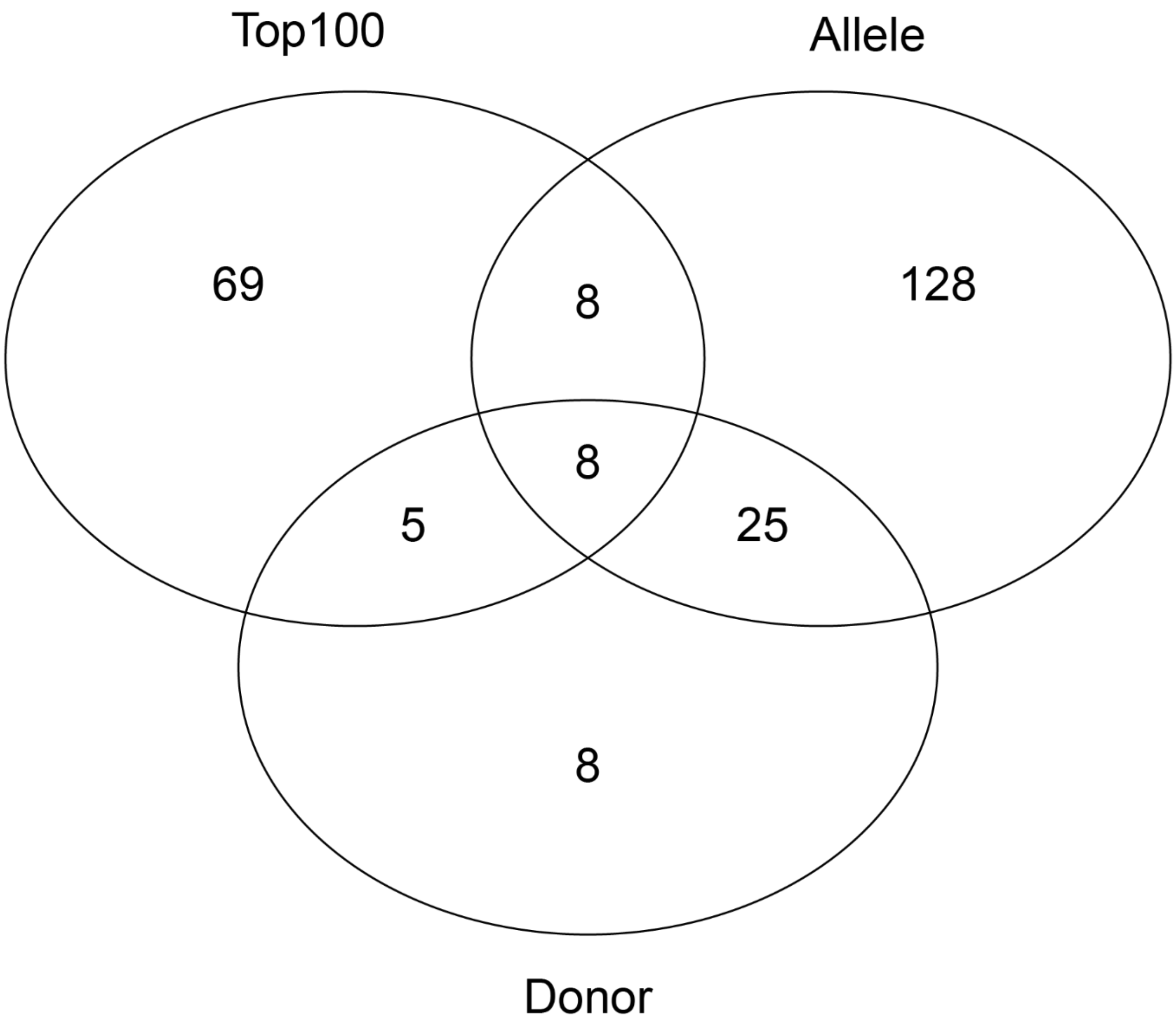

Figure S7. Venn diagram of human universal-peptide source genes originating from the three selection criteria specified in Figure S6 B-D, Related to Figure 4 and Figure 5.

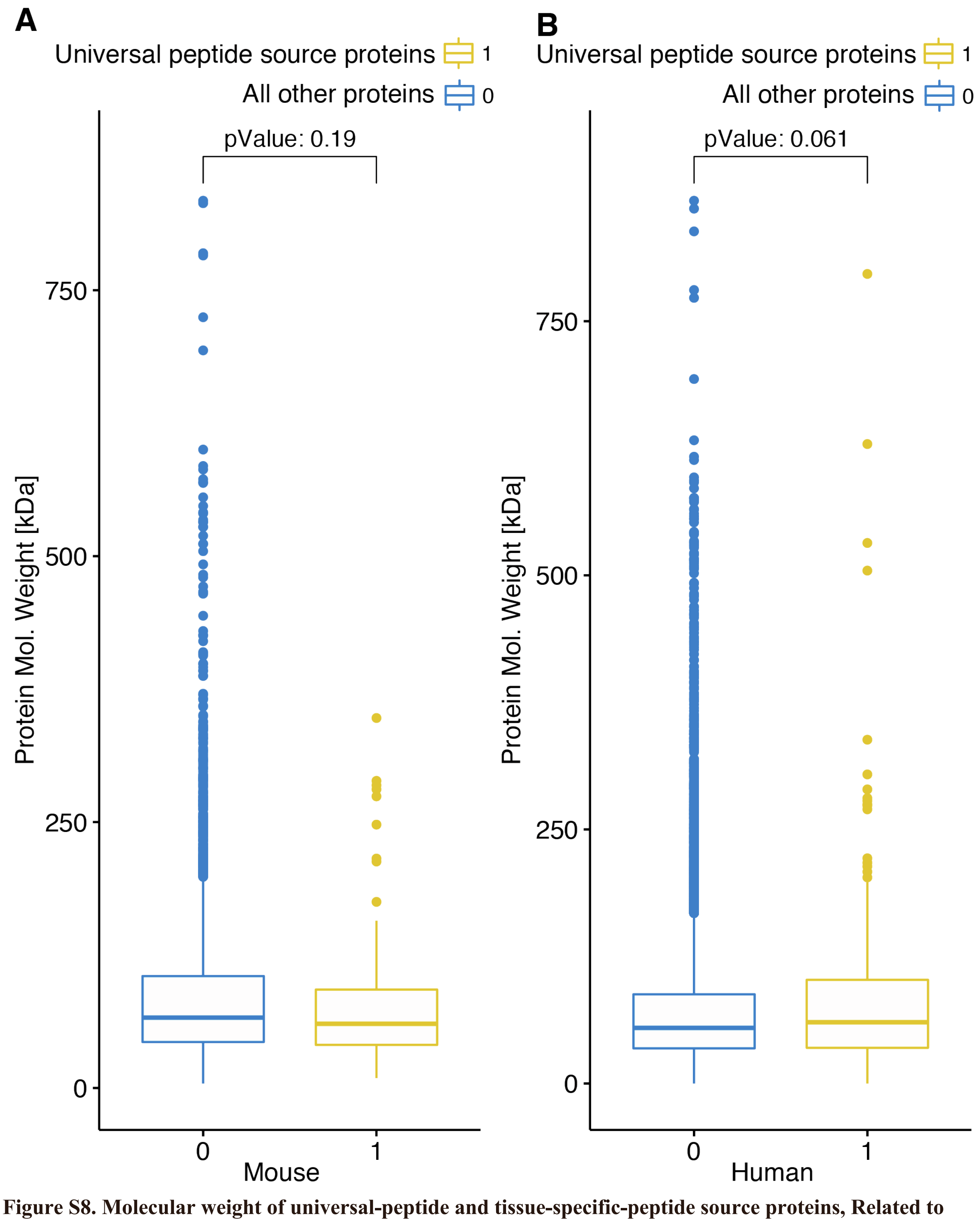

Figure S8. Molecular weight of universal-peptide and tissue-specific-peptide source proteins, Related to Figure 4 and Figure 5. (A) Mouse and **(B)** Human.

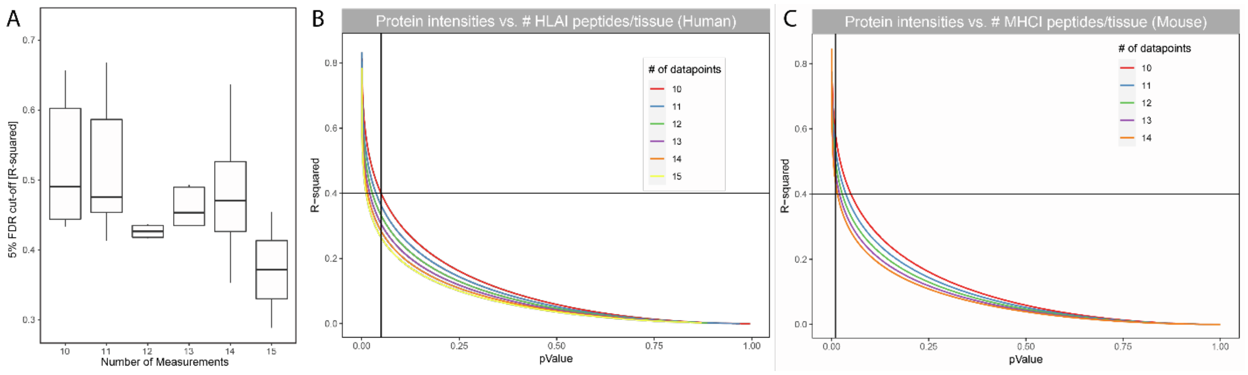

Figure S9. Large scale correlation of protein intensities with the total count of MHCI peptides per tissue in human and mouse datasets, Related to Figure 6. (A) R-squared values of linear fits above which a five percent false discovery rate (5% FDR) threshold is met, computed for each human subject and mouse, individually. This information was used to set an approximate R-squared threshold in addition to the p-value threshold for the linear fits of protein data. **(B)** R-squared of linear fits plotted against the corresponding p-values for the human data. Proteins whose fits show R-squared values> 0.4 (p-value<0.05) in at least two subjects are considered significant. **(C)** R-squared of linear fits plotted against the corresponding p-values for the mouse data. Proteins whose fits show R-squared values> 0.4 (p-value<0.01) are considered significant.

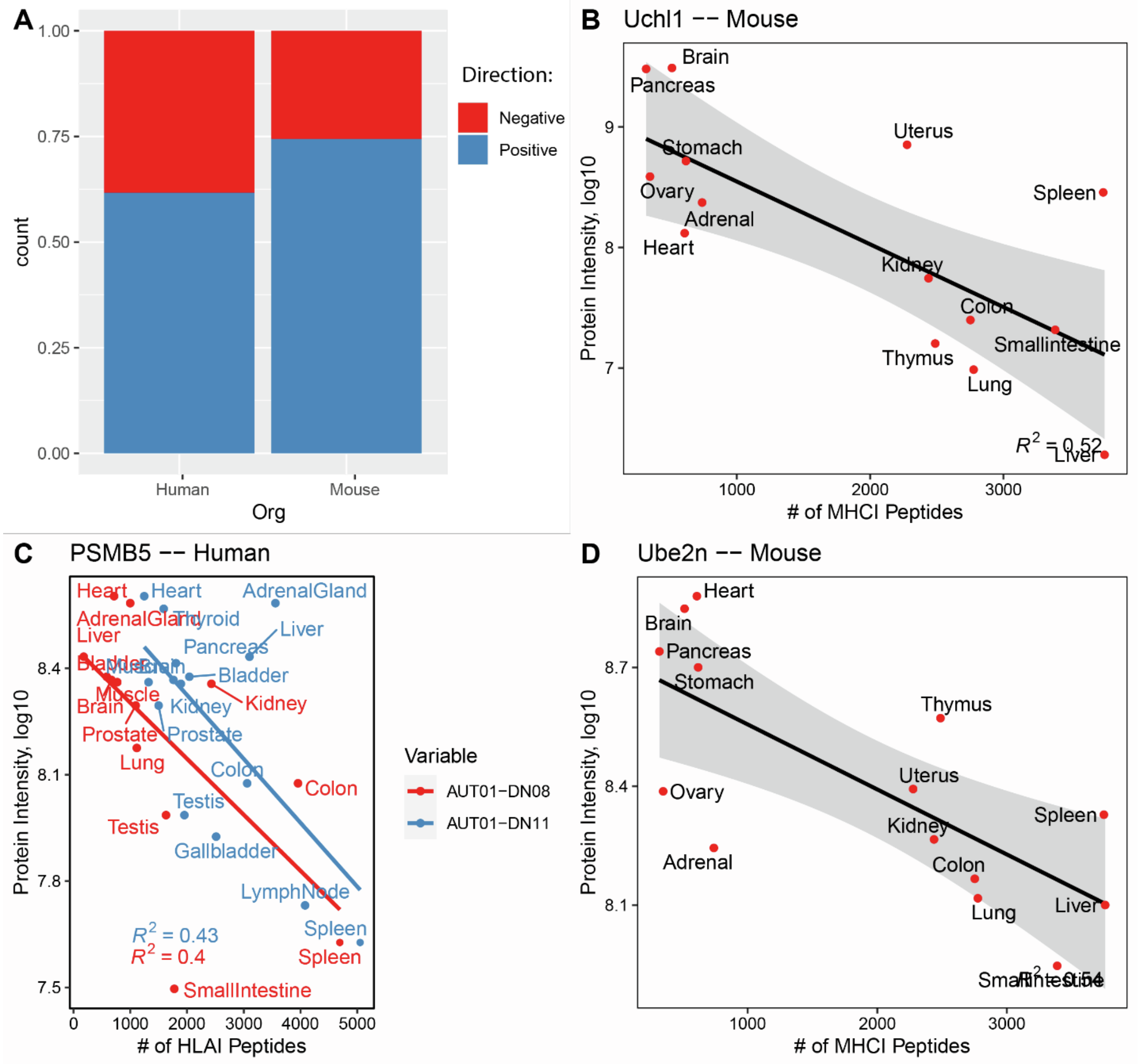

Figure S10. Negative correlations between total MHC-I peptide counts and protein intensities, Related to Figure 6. **(A)** Proportion of direction of slopes of significant proteins in human and mouse. **(B-D)** Example fits of proteins in human and mouse with negative correlation.

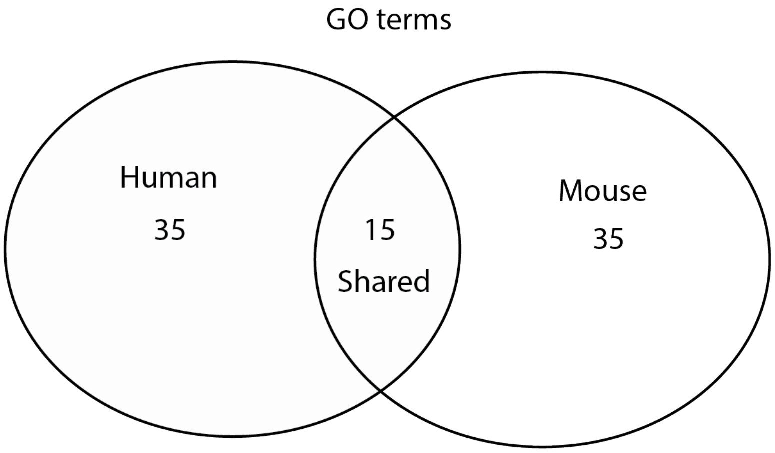

Figure S11. Overlap of enriched gene ontology (GO) terms between Mouse and Human for genes significantly correlating with total MHCI/HLAI counts, Related to Figure 6.

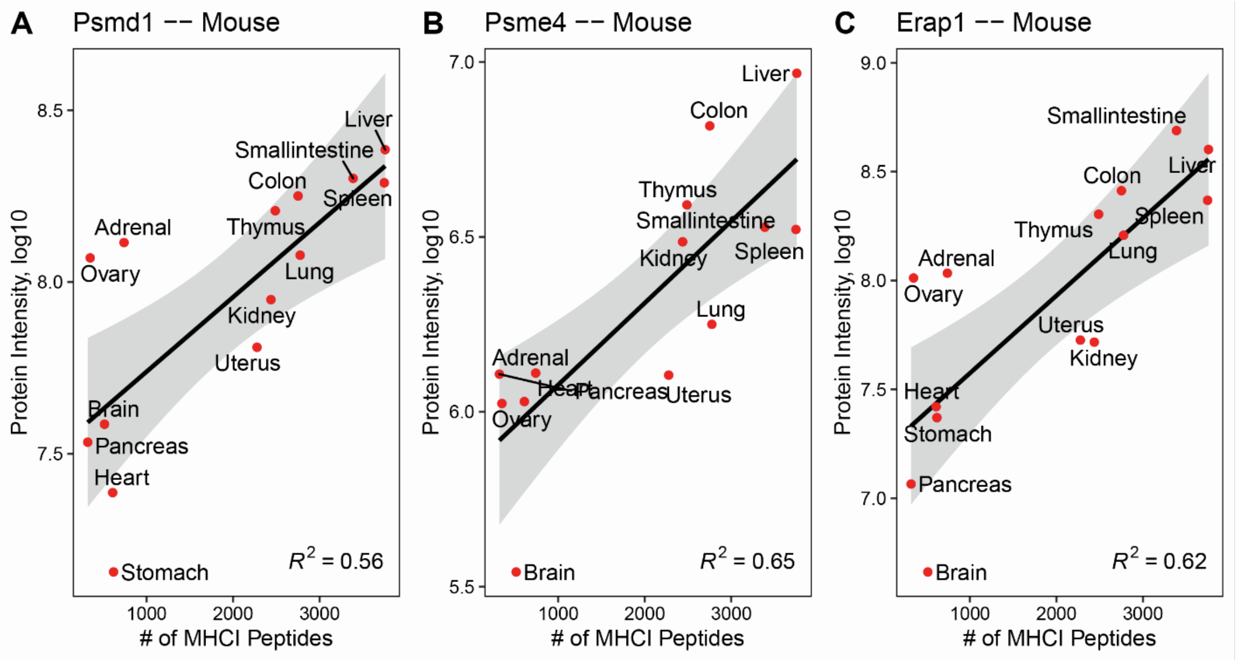

Figure S12. Example fit curves of prominent proteins, Related to Figure 6. (A) Example fit of the protein Esmd1 in mouse. **(B)** Example fit of the protein Psme4 in mouse. **(C)** Example fit of the protein Erap1 in mouse.

Data S1. MHCI atlas user guide, Related to STAR Methods.

MHCIatlas user guide

Peter Kubiniok

2021-03-10

Note to the reader:

This document is a step by step user guide for the R package MHCIatlas. MHCIatlas is intended to allow reproduction of the data-analysis presented in the publication entitled "Global Analysis of the Mammalian MHC class I Immunopeptidome at the Organism-Wide Scale" by Kubiniok et al. This document describes the usage of the R package and presupposes basic knowledge in R. This is not a document that describes and reasons how and why certain analysis were performed in the presented way. Such information can be found in the manuscript itself. Please note that details about each function and embedded parameters (If not already described in this document) can be obtained by running:

```
?MHCIatlas::NameOfFunction
```

For example:

```
?MHCIatlas::MakeCorrMouse
```

1. Install the MHCIatlas R package:

Use devtools install_github function to download and install the MHCIatlas R package directly from GitHub as follows:

```
devtools::install_github('CaronLab/MHCIatlas')
```

In case installation is not possible because dependent R packages are not installed, you can either manually install the missing packages or run the following code:

```
requiredpackages<-c('BiocManager', 'FactoMineR', 'factoextra',
'stringr', 'ggplot2', 'cowplot', 'tidyr', 'stats','broom', 'ggrepel',
'ggpmisc','dplyr', 'magrittr', 'data.table', 'Matrix', 'pheatmap',
'reshape2', 'ggplotify', 'RColorBrewer', 'forcats',
'rtracklayer', 'TxDb.Mmusculus.UCSC.mm10.knownGene',
'TxDb.Hsapiens.UCSC.hg38.knownGene', 'AnnotationDbi',
'org.Mm.eg.db','GenomicFeatures', 'zoo', 'limma',
'webr', 'org.Hs.eg.db', 'data.table', 'scales','ggforce')
for (pkg in requiredpackages) {
  if (pkg %in% rownames(installed.packages()) == FALSE)
  {install.packages(pkg)
    tryCatch({BiocManager::install(pkg)},error=function(e){pkg})}
}
```

And then try the installation again:

```
devtools::install_github('CaronLab/MHCIatlas')
```

2. Load and attach the MHCIatlas R package:

```r
library(MHCIatlas)
```

3. Retrieve human and mouse immunopeptidomics datasets:

Both the human and mouse immunopeptidomics datasets are included in the MHCIatlas package and can be retrieved using the following two functions. Parameters shown are the ones used for the data analysis presented in our manuscript. They can be adjusted as wanted.

```
df_human<- GetHumanMHCIdata(NetMHC_Rank_Threshold = 2,return_all_rawData = FALSE,
                            omitThymus = TRUE)
df_mouse<- GetMouseMHCIdata(NetMHC_Rank_Threshold = 2,return_all_rawData = FALSE)
```

NetMHCpan4.0 thresholds are by default set to 2, meaning that peptides with a predicted Rank score <=2 are considered to be immunopeptides (MHCI binding). Furthermore this functions retrieves a cleaned up version of the search engines output data when return_all_rawData is set to FALSE. The resulting dataframe format is needed to run further functions. When return_all_rawData is set to TRUE, the complete dataset is retrieved. This option should only be used for customized analysis. In human, we ommited the Thymus from the data analysis (omitThymus+TRUE) because it appeared to be an outlier. Thymus samples were gathered from Thymus only donors (Meaning that these donors only donated Thymus samples). This option can be set to FALSE if the reader intends to include the Thymus in the analysis.

4. Generate Figure 2 (Distribution of HLA-A-, B- and C-specific immunopeptidomes across human tissues):

Use the following code to compute the analysis and plots for figure 2:

```
F2<-mkFigure2(df_human)
```

Get the results:

```
print(F2[[2]])
```

The function mkFigure2 retrieves donor specific data from the human immunopeptidomics dataset to plot the proportions of allele representations across tissues (Panels A-D). It also uses the function 'mkHumanConnectivityMap' to calculate the enrichment of over-represented alleles across donors and tissues as is described in the materials and methods section of the accompanying manuscript (Panel E).

5. Generate Figure 3 (Comparison of tissue dependent MHCI (Mouse) and HLAI (Human) peptide antigens):

Figure 3 compares the mouse and human immunopeptidomes. Principal component analysis and the mouse connectivity map are calculated as described in detail in our manuscript.

```
F3<-mkFigure3(df_human, df_mouse)
```

To plot figure 3 use:

```
print(F3)
```

Implemented in this function (mkFigure3) are the following functions:

```
BasicAnalHuman(df_human,df_mouse)
BasicAnalMouse(df_mouse)
```

Both which perform the principal component analysis and retrieve tissue dependent counts of MHCI peptides (Panels A-E). And also the function:

```
mkMouseConnectivityMap(df_mouse)
```

which finds the number of peptides shared between each possible pair of the 19 mouse tissues (Panel F).

6. Generate Figure 4 (mRNA expression of MHCI source genes (Mouse)):

Figure 4 uses the mouse immunopeptidome and mRNA transcriptional data. These datasets are implemented into the MHCIatlas R package.

```
F4<-mkFigure4(df_mouse)
```

To generate the plot for Figure4 use:

```
print(F4)
```

Within the function 'mkFigure4' the mouse transcriptomics data are mapped to the genes in the immunopeptidomics dataset (Panel A). Genes are then classified dependent on their presence in the immunopeptidome. Genes whose immunopeptide signature is found in only one tissue are identified (Panel B) and their mRNA expression across all tissues are compared in the form of z-scores (Panel C).

7. Generate Figure 5 (Expression and genetic conservation of genes coding for MHCI/HLAI peptides presented across most tissues (housekeeping/universal peptides):

```
HumanHousekeepers<- HousekeepersHuman(df_human)
F5<-mkFigure5(
  df_human,
  HumanHousekeepers,
  df_mouse,
  useDefaultCons = TRUE,
  ConsMouse = NA,
  ConsHuman = NA
)
```

The function 'HousekeepersHuman' extracts the human housekeeping genes as described in the manuscript text. The parameters can be changed if wanted. Since it is more straightforward to extract the genes represented by housekeeping (also referred to as universal) immunopeptides, no separate function is needed to obtain mouse housekeeping genes (Those are simply the genes who represent at least one MHCI peptide that has been found across all 19 tissues).

The function mkFigure5 takes the mouse and human housekeeping genes and maps them with the median mRNA expression levels across all tissues in mouse and human (Panels A and B).

Conservation analysis can be performed using the functions 'ConservationHuman' and 'ConservationMouse'. Note however, that 'ConservationHuman' and 'ConservationMouse' can only be run on Linux. The parameter 'useDefaultCons = TRUE' is used to generate panel C and D based on the pre-calculated conservation rates.

To generate Figure 5:

```
print(F5)
```

If the user wishes to calculate conservation rates themselves, 'useDefaultCons = FLASE' has to be used. Conservation rates can than be calculated as follows (Default parameters are shown, those can be adjusted as wanted):

```
ConsHuman<- ConservationHuman(
  df_human,
  HumanHousekeepers,
  pathBW_human = "~/Downloads/hg38.phastCons100way.bw",
  samplesize = 2000,
  quantile = 4,
  ts_DonorSpecific = FALSE,
  MinTissuesPerDonor = 15,
  returnplots = TRUE
)
ConsMouse<- ConservationMouse(
  df_mouse,
  pathBW_mouse = "~/Downloads/mm10.60way.phastCons.bw",
  samplesize = 2000,
  returnplots = TRUE
)
```

Note: R anticipates that the user has downloaded the hg38.phastCons100way.bw (BigWig file human) and mm10.60way.phastCons.bw (BigWig file mouse) and placed them into the 'Downloads' folder of the linux machine. Otherwise a custom path has to be specified.

Once the conservation rates are calculated, the function mkFigure5 can then be run again using the above calculated conservation rates ‘ConsHuman’ and ‘ConsMouse’:

```
HumanHousekeepers<- HousekeepersHuman(df_human)
F5<-mkFigure5(
  df_human,
  HumanHousekeepers,
  df_mouse,
  useDefaultCons = FALSE,
  ConsMouse = ConsMouse,
  ConsHuman = ConsHuman
)
```

And to generate Figure 5 using the custom calculated conservation rates:

```
print(F5)
```

8. Generate Figure 6 panels B-C (Correlation of protein abundances at the proteome-wide scale with the total number of MHCI or HLAI peptides detected across tissues) and compute correlations between protein abundances (proteomics data) and tissue dependent immunopeptide counts:

In order to make Figure6, we first need to generate all correlations between protein intensities and immunopeptide intensities across all tissues. To do so, the two functions 'MakeCorrHuman' and 'MakeCorrMouse' are used. Both use the proteomics datasets from Geiger et al. 2013 (Mouse) and Wang et al. 2019 (Human) which are implemented into the MHCIatlas package as well as the immunopeptidomes. Parameters depicted are the default parameters used to perform the data analysis described in the manuscript. Parameters shown are defaults, they can be changed if wanted.

```
corr_human<- MakeCorrHuman(df_human,Donors = c('all'),
                           deconvolute_byHLAGene = FLASE,pValue_Threshold = 0.05,
                           rsq_Threshold = 0.4,runAnalCorrHuman = TRUE)
corr_mouse<- MakeCorrMouse(df_mouse,pValue_Threshold = 0.01,rsq_Threshold = 0.4,useSILAC = F)
```

As described in the manuscript, significantly correlating proteins were subjected to gene ontology analysis. Gene ontology results are provided within the package. Using the pre-calculated gene ontology results, Figure 6 can be generated as follows:

```
F6<-mkFigure6(corr_human,corr_mouse,GO_human = NA,GO_mouse = NA)
```

The parameters GO_human = NA,GO_mouse = NA can be set to custom data frames that include custom GO terms in the format:

```
#>                             Gene.Set.Name FDR.qvalue
#> 1             GO_INTRACELLULAR_TRANSPORT   1.31e-16
#> 2                         GO_PROTEOLYSIS   1.84e-16
#> 3                           GO_SECRETION   1.17e-15
#> 4 GO_CELLULAR_MACROMOLECULE_LOCALIZATION   2.75e-15
#> 5     GO_MACROMOLECULE_CATABOLIC_PROCESS   5.09e-14
#> 6                          GO_EXOCYTOSIS   6.88e-14
```

Figure 6 can then be plotted by running:

```
HumanHousekeepers<- HousekeepersHuman(df_human)
F6<-mkFigure6(corr_human,corr_mouse,GO_human = NA,GO_mouse = NA)
```

and

```
print(F6)
```

9. Generate plots of singificanlty correlating prteins (Human and Mouse):

In order to visualize correlations computed in step 8, plots for each significantly correlating protein (Mouse and Human) have to be generated. This is done as described below:

```
plots_human <- PlotsHumanProtCorr(
  corr_human,
  SigGene_names = NULL,
  allSigprots = TRUE,
  RankSigThreshold = 2,
  path_filename = NA,
  return_list_of_plots = TRUE
)
plots_mouse <- PlotsMouseProtCorr(
  corr_mouse,
  pValue_Threshold = 0.01,
  SigGene_names = NULL,
  path_filename = NA,
  return_list_of_plots = TRUE
)
```

Now, one can retrieve each single protein plot by subsetting the list of plots with the appropriate gene name. For example:

```
plots_mouse[['Erap1']]
```

And to find all significantly correlating mouse genes:

```
names(plots_mouse)
```

A similar approach works to make plots of the human proteins:

```
plots_mouse[['CD84']]
names(plots_human)
```

Note: You can also provide a 'path/filename.pdf', the function will than generate a large .pdf file with all plots of significantly correlating proteins.

10. Generate Supplementary Figures:

Supplementary Figures can be generated by running the following functions:

```
mkHumanConnectivityMap(df_human)[[2]] #Supplementary Figure 1
SupplFigure2(df_human)
SupplFigure3(df_mouse)
SupplFigure4(df_human)
SupplFigure5(df_human)
SupplFigure6(df_mouse,df_human)
HousekeepersHuman(df_human) #Supplementary Figure 7
SupplFigure10(corr_human, corr_mouse, plots_human, plots_mouse)
```

Supplementary Figure 8 cannot be generated using this R package since proteome wide molecular weight information have to be downloaded from www.uniprot.org directly. This dataset would have been to large to include in this package.

Plots for Supplementary Figure 9 are generated when running the functions 'MakeCorrHuman' and 'MakeCorrMouse'.

Information for supplementary figure 11 can be obtained as shown in the following two code snippets:

Mouse GO terms:

```
read.csv(system.file("extdata", "mouse_GOterms.csv", package = "MHCIatlas"))
```

Human GO terms:

```
read.csv(system.file("extdata", "human_GOterms.csv", package = "MHCIatlas"))
```

The procedure to plot the proteins depicted in supplementary figure 12 is shown in step 9.
